# Sertraline as a new potential anthelmintic against *Haemonchus contortus*: toxicity, efficacy, and biotransformation

**DOI:** 10.1186/s13567-021-01012-x

**Published:** 2021-12-11

**Authors:** Markéta Zajíčková, Lukáš Prchal, Martina Navrátilová, Nikola Vodvárková, Petra Matoušková, Ivan Vokřál, Linh Thuy Nguyen, Lenka Skálová

**Affiliations:** 1 Department of Biochemical Sciences, Faculty of Pharmacy, Charles University, Heyrovského 1203, 500 05 Hradec Králové, Czech Republic; 2 Biomedical Research Centre, University Hospital in Hradec Králové, Hradec Králové, Czech Republic; 3 Department of Pharmacology and Toxicology, Faculty of Pharmacy, Charles University, Hradec Králové, Czech Republic

**Keywords:** Drug repurposing, drug metabolism, hepatotoxicity, drug resistance, nematodes

## Abstract

*Haemonchus contortus* is a parasitic nematode of ruminants which causes significant losses to many farmers worldwide. Since the drugs currently in use for the treatment of haemonchosis are losing their effectiveness due to the drug-resistance of this nematode, a new or repurposed drug is highly needed. As the antipsychotic drug sertraline (SRT) has been shown to be effective against the parasitic nematodes *Trichuris muris*, *Ancylostoma caninum* and *Schistosoma mansoni*, the aim of the present study was to evaluate the possible effect of SRT on *H. contortus*. The potential hepatotoxicity of SRT was tested in sheep, a common *H. contortus* host. In addition, the main metabolic pathways of SRT in *H. contortus* and the ovine liver were identified. While no effect of SRT on *H. contortus* egg hatching was observed, SRT was found to significantly decrease the viability of *H. contortus* adults in drug-sensitive and resistant strains, with its effect comparable to the commonly used anthelmintics levamisole and monepantel. Moreover, SRT in anthelmintically active concentrations showed no toxicity to the ovine liver. Biotransformation of SRT in *H. contortus* was weak, with most of the drug remaining unmetabolized. Production of the main metabolite hydroxy-SRT did not differ significantly between strains. Other minor metabolites such as SRT-*O*-glucoside, dihydroxy-SRT, and SRT-ketone were also identified in *H. contorts* adults. Compared to *H. contortus*, the ovine liver metabolized SRT more extensively, mainly via desmethylation and glucuronidation. In conclusion, the potency of SRT against *H. contortus* was proven, and it should be tested further toward possible repurposing.

**Supplementary Information:**

The online version contains supplementary material available at 10.1186/s13567-021-01012-x.

## Introduction

Since diseases caused by parasitic nematodes are accompanied by various types of clinical complications, the constant and regular control of nematodes infection in livestock is vital for efficient and welfare-friendly production. Pharmacotherapy of animals represents the basic strategy for the treatment of nematodiasis. Four major classes of common anthelmintics are routinely administered to livestock: benzimidazoles (e.g. mebendazole, albendazole, fenbendazole, flubendazole), imidazothiazoles (levamisole), macrocyclic lactones (ivermectin, moxidectin), and the amino-acetonitrile derivative monepantel [1, 2].

Nevertheless, the effectiveness of the available anthelmintics over time has become limited due to increasing drug resistance in nematode populations [3]. The development of variable degrees of drug resistance among nematodes has been reported for all groups of anthelmintics [3] and the prevalence of resistance has increased globally due to massive drug administration [4, 5]. The fact that resistance to monepantel, the most recent anthelmintic in wide use, has occurred within less than four years of the product first being introduced is certainly disturbing [6, 7]. Therefore considerable efforts have been devoted to the development of new anthelmintic drugs, especially those with more pronounced efficacy in nematodes resistant to classical anthelmintics [8].

Several approaches are typically used to identify a new anthelmintic [9]. Among these, drug repurposing is an interesting strategy based on testing drugs approved for the treatment of other diseases for anthelmintic activity. The major advantage of this strategy, also called “therapeutic switching”, is that a great deal of information about these drugs is already well known, and a new candidate may be made ready for clinical trials relatively quickly [13]. Over the past years, a variety of drug repurposing initiatives have been launched against many diseases, including helminthiases [13].

Concerning anthelmintics, broadening the range of target helminth species or treated organisms represents the most basic strategy. In addition, new anthelmintics might be found among drugs or other compounds with totally different indications of use. A screening of a small-molecules library of compounds used in human clinical trials against the model nematode *Caenorhabditis elegans* uncovered the anthelmintic activity of the neuromodulatory drugs sertraline (SRT), paroxetine and chlorpromazine [10]. Subsequently, these FDA-approved drugs showed significant anthelmintic activity against three nematode species in lower effective concentrations: they decrease motility of adult *Trichuris muris* whipworms, prevent hatching and development of *Ancylostoma caninum* hookworms, and kill *Schistosoma mansoni* flatworms [10]. *C. elegans* mutants with resistance to known anthelmintics are also susceptible to these drugs, suggesting that they may act on novel targets to kill worms. Comparing the efficacy of these three drugs, SRT seems to be the most promising [10].

With respect to these findings, the present study was designed to test the efficacy of SRT against the nematode *Haemonchus contortus*, a widespread parasite with high prevalence of drug-resistance. Two *H. contortus* strains with a different sensitivity to classical anthelmintic drugs were used in the study: ISE (Inbred-Susceptible-Edinburgh, MHco3) strain, which is sensitive to all classes of anthelmintics and IRE (Inbred-Resistant-Edinburgh; MHco5) strain with decreased sensitivity to ivermectin (IVM) and benzimidazole drugs [11, 12]. Besides the classical egg hatch test, the effect of SRT in *H. contortus* adults was also monitored using an ATP bioluminescent assay [13]. The efficacy of SRT in *H. contortus* adults was compared with the commonly used anthelmintic drugs levamisole (LEV) and monepantel (MOP). In addition, SRT biotransformation in *H. contortus* was studied to reveal the ability of this parasite to protect against SRT via its deactivation. As the sheep are the intended target species, SRT potential toxicity and biotransformation has also been analysed in the ovine liver.

## Materials and methods

### Chemicals

Monepantel (MOP) was purchased from Toronto Research Chemicals (Canada). Ethyl acetate (HPLC grade), acetonitrile (ACN, LC–MS grade) and ethanol were obtained from VWR International s.r.o. (Stříbrná Skalice, Czech Republic). Pierce™ BCA Protein Assay Kit and Collagenases (Gibco) was purchased from Thermo Fisher Scientific (Prague, Czech Republic). Sertraline hydrochloride (SRT), RPMI-1640 medium (Roswell Park Memorial Institute medium), Williams’ medium E powder without sodium bicarbonate, Ham’s Nutrient Mixture F12 (HAM F12), Penicillin–Streptomycin (Pen-Strep, 10 000 U Pen and 10 mg Strep / mL), and all other chemicals were obtained from Sigma–Aldrich (Prague, Czech Republic). Ultrapure water of ASTM I type (resistance 18.2 MΩ.cm at 25 °C) was prepared by the Barnstead Smart2Pure 3 UV/UF apparatus (Thermo Fisher Scientific, Bremen, Germany).

### Collection of biological material

Biological materials for the experiments including various stages of *H. contortus* (eggs and adults) and ovine liver were obtained from 6-month old lambs. The lambs were firstly dewormed by a single dose of albendazole (*per os* individually, 5 mg/kg), then orally infected with 8000 larvae L3 of *H. contortus* of ISE or IRE strain. Three lambs were slaughtered for one biological replicate from each strain.

The eggs were isolated from feces collected daily four weeks post-infection (pi). The fecal pellets were firstly manually homogenized in tap water, then the homogenate was passed through three sieves with decreasing mesh diameter (250 µm, 100 µm, 25 µm). The first two sieves remove coarse particles and the last one serves for capturing the eggs. From the last sieve the mass containing the eggs was rinsed and transferred into 50 mL falcon tube and centrifuged (Centrifuge Eppendorf 5810R, 1600 rpm (481 × *g*), 3 min). The supernatant was removed and sediment was resuspended in flotation solution (FS, saturated sucrose solution with density 1.27 g/cm^3^) and again centrifuged (1000 rpm (188 × *g*), 3 min), following which the upper layer containing eggs was transferred into a new 15 mL falcon tube and topped up by fresh FS, then again centrifuged (1000 rpm, 3 min). The upper layer was again transferred into a new falcon tube and topped up with tap water and centrifuged (1600 rpm, 3 min). After centrifugation, the eggs are seated at the bottom of the flask. To remove the rest of the FS and dirt, the eggs were rinsed 5 times with tap water, following which the clean eggs were used for the egg hatch tests.

Six weeks pi the lambs were slaughtered, and the abomasa and liver were removed. The abomasa were kept in warm water (37 °C) and transported to the laboratory, where the adults were harvested using the agar method [14], followed by manual separation of males and females. One lobe of the liver was perfused with chilled Euro Collins solution and transported to the laboratory in a chilled vessel.

The animal protocols comply with the Guide for the Care and Use of Laboratory Animals (Protection of Animals from Cruelty Act No. 246/92, Czech Republic). All experimental procedures were evaluated and approved by the Ethics Committee of the Ministry of Education, Youth and Sports (Protocol MSMT-25908/2019).

### Egg hatch test

Freshly isolated eggs of *H. contortus* were incubated for 48 h in 96 well plates with increasing SRT concentrations which were two-fold serially diluted in dimethyl sulfoxide (DMSO) into 9 concentrations. 1 µL was pipetted into a 96-well flat bottom plate, and 199 µL water solution of eggs (50 eggs/well) was added. The final concentration of anthelmintics was 0, 0.8, 1.6., 3.1, 6.3, 12.5, 25.5, 50, 100 and 200 µM. The controls contained 50 eggs in 199 µL of water and 1 µL of DMSO. The plates were incubated at 27 °C and stopped by adding 5 µL of Lugol’s iodine after 48 h. The proportion of unhatched eggs and larvae was counted under a microscope. IC_50_ was calculated using GraphPad Prism 9.1.2.

### Viability test of *H. contortus* adults

The effect of SRT on *H. contortus* was ascertained by measuring ATP content in the adult worms [13]. Similarly, adults of *H. contortus* were incubated with the commonly used drugs MOP and LEV.

The adults of *H. contortus* (males and females separately) were incubated with increasing concentrations of the tested compound pre-dissolved in DMSO in supplemented RPMI 1640 medium (Roswell Park Memorial Institute medium) for 48 h using 24 well plates. The final concentrations of SRT were 0, 1, 10, 20, 30, 40, 50 µM and the final concentration for LEV and MOP were 0, 1, 10, 20 µM. One well contained 900 µL of media with the tested compound (or 0.1% DMSO in the controls) and 4–8 females or 8–16 males. The medium was supplemented with 0.8% glucose, 0.25 µg/mL amphotericin B, 10 U/mL penicillin, 10 µg/mL streptomycin and 10 mM HEPES [15]. After incubation the worms were first washed in PBS (phosphate buffered saline tablets, pH 7.2–7.6), then 1 female or two males were placed separately into 2 mL tubes containing 50 µL of SONOP (sonification solution, consisting of 70% ethanol with 2 mM EDTA (ethylenediaminetetraacetic acid)), rapidly frozen in dry ice, and stored in the freezer (−80 °C) until measurement.

To measure the ATP level, 450 µL of chilled Tris/EDTA buffer (100 mM Tris, 2 mM EDTA, pH adjusted by HCl to 7.6–8.0) was added to each sample. The samples were homogenized 30 s (6 m/s) in FastPrep-24 homogenizer (MP Biomedicals, Santa Ana, CA, USA) then centrifuged for 10 min (Thermo Scientific Biofuge Stratos, 13 200 rpm (16 978 × *g*)) at 4 °C. ATP level content was measured by the ATP Bioluminescence Assay Kit CLS II (Roche, Mannheim, Germany) according to the manufacturer’s protocol, with adjustments described in [13].

To eliminate variability in worm size in the adults, the ATP concentration was related to µg/mL of total proteins in a sample pellet. The protein was measured in technical duplicates per sample using bicinchoninic acid based on the manufacturer’s protocol (PierceTM BCA Protein Assay Kit, ThermoScientific), with adjustments described in [13].

### Hepatotoxicity tests

Two methods were used to test the hepatotoxicity of SRT in the ovine liver, the first was based on the measurement of ATP level in precision-cut liver slices, the second was based on the reduction of MTT (3-(4,5-dimethylthiazol-2-yl)-2,5-diphenyltetrazolium bromide) in a primary culture of isolated hepatocytes.

The preparation of liver slices and the measurement of ATP levels was performed according to Zárybnický, Matoušková [16]. The slices were incubated in supplemented Williams’ Medium E with increasing concentrations of SRT (0, 1, 10, 50, 100 µM, pre-dissolved in DMSO) for 24 h. Control slices were incubated in medium with 0.1% DMSO only. The medium was supplemented with glucose (final concentration 36 mM) and gentamycin 50 μg/mL. After incubation, the slices were gently collected, washed in PBS and then placed into 150 μL of SONOP and snapped frozen in dry ice. The samples were kept in a freezer (−80 °C) until measurement. For ATP level measurement, the slices were firstly homogenized (FastPrep homogenizer, 6 m/s, 20 s), then centrifuged for 5 min (centrifuge Eppendorf, 13 200 rpm (16 978 × *g*)). Prior to centrifugation 350 μL of chilled SONOP was added into each sample. ATP level content was measured by the ATP Bioluminescence Assay Kit CLS II (Roche, Mannheim, Germany) according to manufacturer’s protocol.

For isolation of the hepatocytes, a two-step collagenase method was used, i.e. a piece of liver was firstly perfused by EGTA (ethylene glycol-bis(β-aminoethyl ether)-N,N,N′,N′-tetra acetic acid) containing solution (0.14 mg/mL), then perfused by collagenase solution (1 mg/mL) [17]. The viability of the hepatocytes was tested using a Trypan Blue exclusion assay (Trypan Blue solution 0.4%). Only hepatocytes with viability >75% were used for the experiments. Isolated hepatocytes suspended in culture medium [18, 19] were seeded into 96 well plates precoated with collagen, with the density of the hepatocytes at 50 000 cells per well. After four hours of pre-incubation in a humid atmosphere with 5% of CO_2_ at 37 °C, the hepatocytes were treated with culture medium containing SRT (0, 1, 10, 25, 50, 75, 100 µM pre-dissolved in DMSO) and incubated for 24 h in the same conditions. The final concentration of SRT was 0–75 µM. Control samples contained culture medium with 0.1% DMSO. After incubation, 25 µL of MTT solution dissolved in 1 mL of 0.1 M phosphate buffer (pH 7.4) at a concentration of 3 mg/mL was added into each well and incubated for 1–2 h. When the formazan crystals were visible, the medium was replaced by 50 µL of solubilization solution (0.08 M HCl in isopropanol) and incubated at 37 °C for 30 min. Absorbance was measured at 570 nm (Spark Control Tecan v. 2.2).

### Determination of SRT biotransformation in ***H. contortus*** adults

Adults of *H. contortus* were incubated in 24 well plates (15 males or 10 females in one well with 1.5 mL of RPMI 1640 media supplemented as described previously) for 24 h at 37 °C in a humid atmosphere 5% CO_2._ Blank biological sample (control) contained 0.1% DMSO. Concentration of SRT was 10 µM. After incubation, the worms were washed three times with phosphate buffer saline and transferred into plastic tubes. Medium was also collected and placed into the plastic tubes. The samples were frozen at -20 °C and stored until extraction.

### Determination of SRT biotransformation in ovine liver

SRT metabolites formed in ovine liver were identified using two models: isolated hepatocytes and precision cut liver slices prepared in the same way as for hepatotoxicity tests.

Prepared liver slices (after 2-h pre-incubation) were placed into supplemented Williams’ Medium E medium containing 10 µM SRT pre-dissolved in DMSO and incubated for 24 h. Blank slices were incubated in medium with 0.1% DMSO only. After incubation, the slices were washed in PBS and placed into a 1.5 mL microtube with 200 µL of ultra-pure water and stored at −20 °C until extraction and analyses. Medium was also collected and stored under the same conditions.

Isolated hepatocytes were seeded into a petri dish (6 cm diameter) precoated with collagen, with the density of the hepatocytes 3 × 10^6^ per dish. After four hours of pre-incubation in a humidified atmosphere with 5% of CO_2_ at 37 °C, the hepatocytes were treated with medium containing SRT (10 µM) pre-dissolved in DMSO and incubated for 24 h in the same conditions. Blank samples contained 0.1% DMSO. After incubation, the medium and hepatocytes were collected separately and stored at −20 °C until extraction and analyses.

### Extraction procedures prior to analysis

For the extraction of liver slices, hepatocytes, and *H. contortus* adults the two-step liquid–liquid extraction (LLE) was used. Firstly, the volume of the samples was topped up to 1 mL with ultra-pure water, then the samples were homogenized in FastPrep homogenizer (6 m/s, 30 s) using zirconia beads (sizes of 1.0 mm: 1.4 mm: 2.0 mm in 1: 1: 0.5 ratio, approx. 0.75 g). After homogenization, 900 µL of the sample was transferred into a 5 mL plastic tube. The remaining 100 µL of the sample was evaporated (10 h, 45 °C, concentrator Eppendorf), and used for the evaluation of protein content. 1 mL of media was also transferred into 5 mL plastic tubes, following which 3.4 µL of internal standard (IS, D3-SRT, methanol solution 100 µg/mL) and 1.8 µL of ethanol (to improve extraction efficacy [20]) was added into each sample. The samples were shaken for 1 h with 3 mL of ethyl acetate. After centrifugation (Centrifuge Eppendorf 5810R, 3 min, 3000 rpm (1690 × *g*)), 2.7 mL of upper organic phase supernatant was replaced with fresh ethyl acetate and the extraction process was repeated. Supernatants from both extractions were put together, evaporated to dryness (Concentrator Eppendorf plus, Hamburg, Germany, 30 °C) and stored in a refrigerator (4 °C) until UHPLC-MS analyses. Prior to the analysis, the samples were reconstituted in 100 µL of 30% (v/v) ACN and filtered through syringe filters (Polytetrafluoroethylene, PTFE, 4 mm, 0.22 µm, pk/1000).

### Protein content measurement to determine the biotransformation of SRT in ovine liver and *H. contortus* adults

Protein content was quantified using bicinchoninic acid assay in a 96 well plate according to manufacturer’s protocol with the following adjustments. The samples were firstly evaporated in a concentrator (Eppendorf, 45 °C), after which the remaining pellet of the worm samples or liver samples was incubated with 5 M NaOH for 1 h, 800 rpm at 37 °C (Thermomixer Comfort, Eppendorf) followed by dilution in distilled water to a final concentration of 1 M NaOH. The calibration curve was prepared by serial dilutions of BSA (Bovine serum albumin) in 1 M NaOH.

### UHPLC-HRMS/MS analysis

The UHPLC system (Dionex Ultimate 3000) equipped with a HRMS detector (Q Exactive Plus Orbitrap mass spectrometer, Thermo Fisher Scientific, Bremen, Germany) was used to identify SRT metabolites and acquire their MS/MS spectra. The system consists of the RS LPG Quaternary Pump, RS Column Compartment, RS Autosampler and Chromeleon software (version 7.2.9, build 11,323; Thermo Fisher Scientific, Germering, Germany). Thermo Xcalibur software (version 4.3.73.11) was used for the analyses.

The chromatographic conditions were as follows: Column Zorbax Eclipse Plus C18 (2.1 × 150 mm, 1.8 µm, Agilent, Santa Clara, California, USA); column temperature 35 °C, mobile phase A – ASTM I type ultrapure water containing 0.1% (v/v) formic acid (LC–MS LiChropur™, 97.5–98.5%), mobile phase B – ACN containing 0.1% (v/v) formic acid; flow rate of the mobile phase at 0.3 mL/min. To separate the SRT and its metabolites, a gradient chromatographic method was used as follows: 0.0–1.0 min. – 10% B; 1.0–7.0 min. – increase to 40% B; 7.0–11.0 min. – increase to 100% B; 11.0–12.0 min. – B kept at 100%; 12.0–17.0 min – B kept at 10% B to equilibrate the column before the next sample injection. The total run time was 17 min. From each sample 5 µL was injected into the column.

HRMS and HRMS/MS analyses were performed in positive mode for all samples. The settings of the heated electrospray source were as follows: spray voltage – 3.5 kV; capillary temperature—262.5 °C; sheath gas – 50 L/min; drying gas – 12.5 L/min; nebulizing gas—2.5 L/min; probe heater temperature – 400 °C; max spray current – 100 µA; S-lens RF Level – 50. Total ion current spectra were collected at resolution 140,000 in the range of 105–1000 m*/z*. To determine MS/MS spectra for potential metabolites, parallel reaction monitoring was performed at normalized collision energy 20. The compounds found only in the incubated samples but not in the chemical and biological blank samples were considered to be SRT metabolites and subjected to further analysis. The metabolites were identified and tentative structures proposed based on the accurate mass and MS/MS spectra of the precursor ion.

### UHPLC- MS analysis

Once the metabolites were identified, confirmation and semi-quantification was performed using the triple quadrupole mass analyzer (LCMS-8030, Schimadzu, Kyoto, Japan) connected to the UHPLC Nexera liquid chromatograph (Shimadzu, Kyoto, Japan). The column used and mobile phase were the same as for the UHPLC-HRMS. The chromatographic conditions were as follows: column temperature 40 °C; flow rate of the mobile phase 0.4 mL/min; method started in 0.0 min with 20% of B, followed by linear gradient to 100% of B in 8.0 min and remaining constant to 10.0 min. Between 10.1 and 12 min. B was set back to 20% then equilibrated for 2 min. before the next sample injection. The total run time was 14 min. From each sample 1 µL was injected into the column.

The electrospray parameters for mass spectrometry analysis were as follows: Spray voltage – 4.5 kV; Capillary temperature – 250 °C; drying gas – 13 L/min; nebulizing gas – 2.5 L/min; heat block temperature – 400 °C. Analysis was performed in positive ion mode.

Individual compounds were identified based on the monitoring of selected ion transitions (selected reaction monitoring, SRM). The specific SRM conditions for SRT and D3-SRT were optimized through direct injection of the standard solution into the instrument. For data analyses LabSolution LCMS software 5.93 (Shimadzu, Kyoto, Japan) was used.

### Statistical analysis

The reported data in all the experiments are presented as the mean ± S.D. For statistical analysis, (SA) GraphPad Prism 9.1.2 software was used. The results with *P* < 0.05 were considered significant. Data for the viability of *H. contortus* measured by the ATP method were obtained from two independent experiments with 4–8 biological replicates in each experiment, the outliers (due to the variability of the living system) were removed based on initial data analysis (SA: One-way ANOVA with Dunnett’s multiple comparison test to evaluate concentration dependency on viability, and two-way ANOVA with Šídák’s multiple comparison test to compare strains and gender). For analyzing data from the hepatotoxicity tests and egg hatch tests, one-way ANOVA with Dunnett’s multiple comparison test was used. Data measured by the MTT method were obtained in four independent experiments with six technical replicates. Data for hepatotoxicity test measured by the ATP method were obtained from four independent experiments in 2–4 technical replicates. Data for egg hatch tests were performed in three independent experiments with 2 technical replicates. Concentration of ATP was calculated from the linear regression equation and was normalized to mg of protein. The data from MTT were normalized to control (0.1% DMSO), which represents 100% viability. Data for metabolites identification and semi-quantification were performed in three independent experiments with three replicates (SA: two-way ANOVA with Tukey’s multiple comparison test to compare strains, and Šídák’s multiple comparison test to compare gender). The semi-quantification of metabolites in homogenates was performed by peak area ratio between the metabolite and IS normalized to mg of protein.

## Results

### Effect of SRT on ***H. contortus*** egg hatching

**Figure 1:**
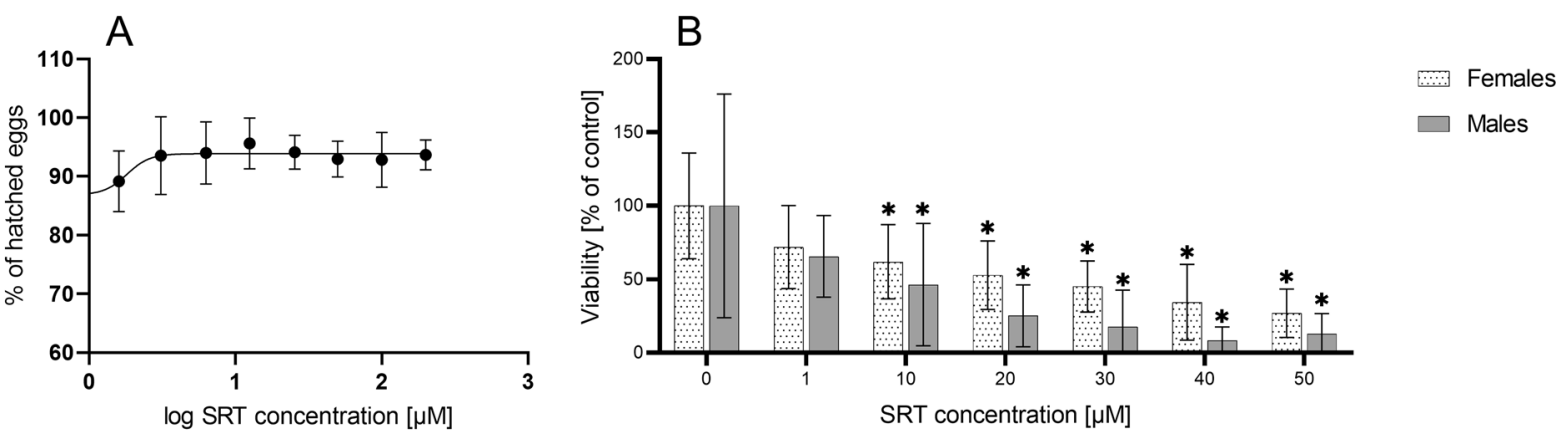
**Effect of SRT of *H. contortus* (ISE strain) eggs hatching (A) and adults (B) viability.** Data are presented as means ± SD (*n* = 3). Statistical analysis was performed by One-way ANOVA with Dunnett’s multiple comparison test. The * marks statistical significance in comparison to control (*P* < 0.05). The control samples were incubated with 0.1% DMSO.

The effect of SRT (at concentrations 0–200 µM) on the hatching of the *H. contortus* eggs was tested and the results presented in Figure 1A. However, no effect of SRT on the *H. contortus* eggs was detected.

### Effect of SRT on the viability of *H. contortus* adults

**Figure 2:**
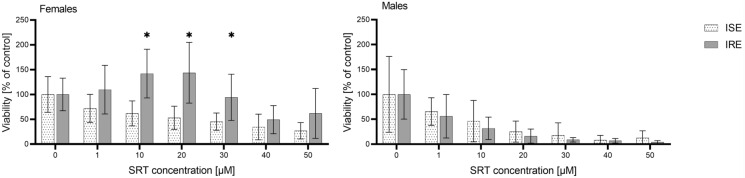
**Comparison of SRT effect in *H. contortus* females and males of ISE and IRE strains.** Data are presented as mean ± SD (*n* = 12). Statistical analysis was performed by Two-way ANOVA with Šídák’s multiple comparison test. The * marks statistical significance *P* < 0.05 in comparison between the strains. The control samples were incubated with 0,1% DMSO.

The effect of SRT (at concentrations 0–50 µM) was tested separately in males and females of the ISE strain and IRE strain of *H. contortus* by measuring the ATP level. In both genders of the ISE strain, SRT significantly decreased the viability of the nematodes in a concentration-dependent manner. The males, however, were more sensitive to SRT than the females (see Figure 1B). A comparison of the SRT effect on the ISE and IRE strains is demonstrated in Figure 2. In females of the IRE strain, SRT increased the ATP level up to concentration 20 µM. A significantly decreased viability (level of ATP) in females of the IRE strain was observed only at concentrations 40 and 50 µM. In the males, SRT decreased the viability in the IRE strain similarly to the ISE strain. The calculated IC_50_ values of SRT for both genders and both strains are presented in Table 1.

**Table 1:** **The IC**_**50**_** values (µM) of SRT for males (M) and females (F) of *H. contortus* ISE and IRE strains (*n* = 12)**

	ISE F	ISE M	IRE F	IRE M
IC_50_ (mean ± SD)	15.90 ± 1.32	4.15 ± 1.55	49.40 ± 1.14	1.98 ± 1.40
IC_50_ (95% CI)	9.13–27.71	1.73–9.97	38.04–64.14	1.03–3.82

The data are presented as means ± standard deviation (SD) and 95% confidence interval (CI).

**Figure 3:**
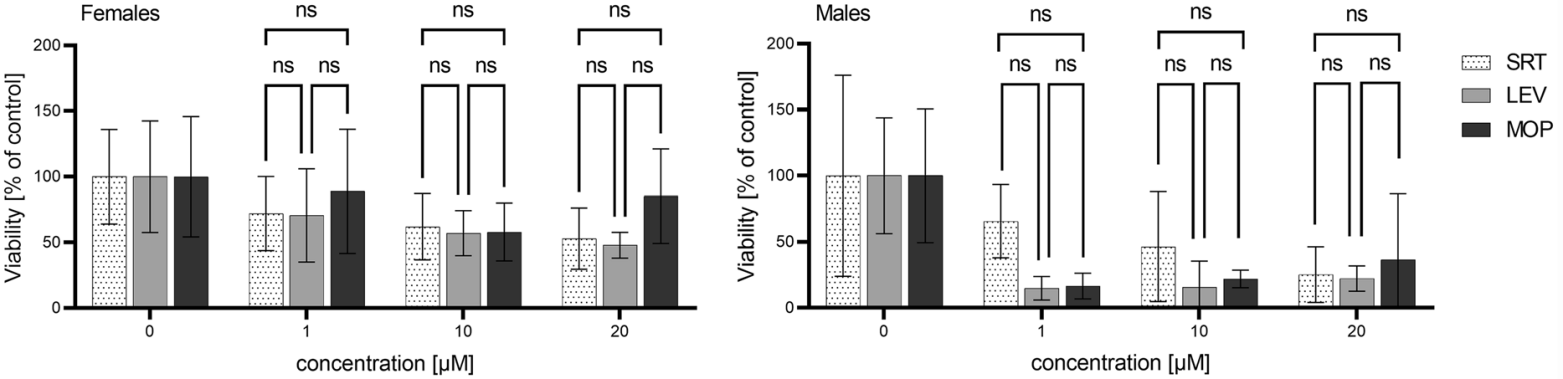
**Comparison of effect of MOP, SRT and LEV on viability of females and males in *H. contortus* ISE strain**. Data are presented as mean ± SD (*n* = 4). Statistical analysis was performed by Two-way ANOVA with Tukey’s multiple comparison test. The control samples were incubated with 0.1% DMSO.

Additionally, the effect of SRT and the two commonly used anthelmintic drugs LEV and MOP on the *H. contortus* ISE strain adults was compared (see Figure 3). In the females, the effect of all three anthelmintics was similar. In the males, although LEV and MOP seemed to be more effective, the differences were not statistically significant.

### Effect of SRT in ovine liver

**Figure 4:**
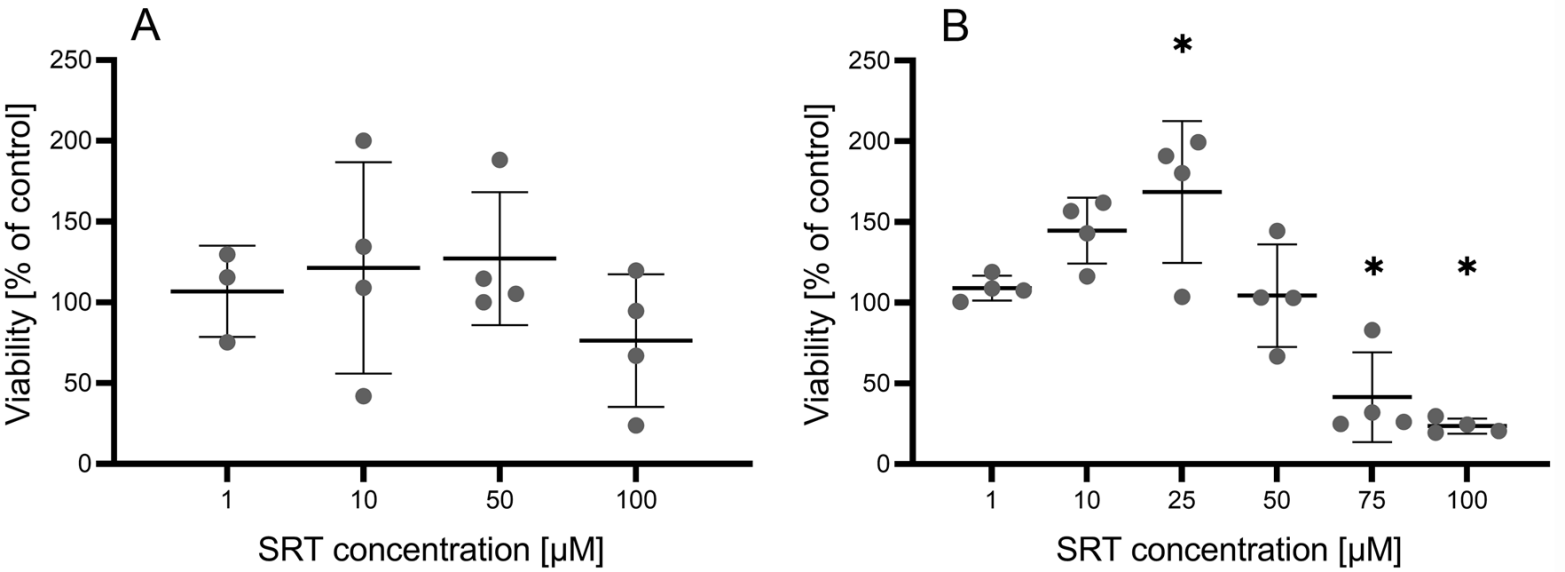
**Effect of SRT on viability of precision cut liver slices (A) and isolated hepatocytes (B).** Data are presented as means ± SD (*n* = 4). Statistical analysis was performed by One-way ANOVA with Dunnett’s multiple comparison test. The * marks statistical significance in comparison to control (*P* < 0.05). One dot represents average from technical replicates in one experiment.). The control samples were incubated with 0.1% DMSO.

To test the potential hepatotoxicity of SRT, two in vitro models were used: precision-cut liver slices and a primary culture of isolated hepatocytes, with the results presented in Figure 4. SRT at concentrations up to 100 µM did not significantly decrease viability in the liver slices. In the hepatocytes, 25 µM SRT increased the viability, while 75 and 100 µM SRT decreased the viability.

### Biotransformation of SRT in *H. contortus* adults

Females and males of the ISE and IRE strains were incubated ex vivo with SRT (10 µM) for 24 h. The metabolites formed were identified based on their accurate masses and MS/MS spectra using HRMS, after which semi-quantification was performed using a triple quadrupole mass analyzer. A list of the metabolites with the accurate masses, retention times and fragments of the metabolites is presented in Table 2. The extracted ion chromatograms and MS/MS fragmentation spectra can be found in Additional files 1, 2, 3, 4, 5, 6, 7, 8, 9 and 10.

**Table 2:** **List of the main metabolites, SRT and D3-SRT detected in samples of *H. contortus* with their retention times (t**_**R**_**) from LC–MS and LC-HRMS, m/z of precursor and product ions detected by LC-HRMS, elemental composition and designation**

Compound	Elemental composition	t_R_ LC–MS [min]	t_R_ LC-HRMS [min]	*m/z* precursor ions [M + H]^+^	*m/z* product ions [M + H]^+^	Designation
Hydroxy-SRT	C_17_H_17_Cl_2_NO	3.74 4.04	10.49 ^1^ 11.36 ^2^	322.0760	304.0661 ^2^ 291.0338^1^ 273.0233 ^1, 2^ 238.0542 ^1, 2^	SRT-OH
SRT-O-glucoside	C_23_H_27_Cl_2_NO_6_	3.14 3.57	9.54 ^1^ 10.65 ^2^	484.1285	273.0232 ^1, 2^ 194.1024 ^1, 2^ 176.0918 ^1, 2^	SRT-O-GLC
Dihydroxy-SRT	C_17_H_17_Cl_2_NO_2_	3.18 3.34	9.74 ^1^ 10.02 ^2^	338.0717	320.0599 ^1^ 289.0185 ^1, 2^ 261.0230 ^1, 2^ 247.0077 ^1, 2^	SRT-2OH
SRT-ketone	C_16_H_14_Cl_2_O	3.51	10.48	291.0338	273.0233 238.0543 145.0649	SRT=O
SRT	C_17_H_17_Cl_2_N	4.29	12.13	306.0810	275.0387 158.9762 129.0699 91.0548	
D3-SRT (IS)	C_17_H_17_Cl_2_N	4.29	12.13	309.0991	275.0394 158.9766 129.0699 91.0548	

The parent compound SRT with *m/z* 306.08 [M + H]^+^ was eluted at 12.13 min, resulting in product ions *m/z* 275.04, 158.99, 129.07 and 91.06. *H. contortus* does not metabolize sertraline very extensively, thus most of the parent drug remained unmetabolized. Two positional isomers of hydroxy SRT (SRT-OH) at *m/z* 322.08 [M + H]^+^ found at t_R_ 10.48 and 11.36 min were the main metabolites identified in the *H. contortus* adults. The most dominant product ion in both metabolites was *m/z* 273.02. Other important product ions observed in fragmentation spectra were *m/z* 304.07, 291.03 and 238.05. The product ion *m/z* 304, which represents the neutral loss (NL) of water, was detected only at t_R_ 11.36 min, while the product ion *m/z* 291 was detected only at t_R_ 10.48 min. The product ion *m/z* 238, the radical that resulted from the loss of chlorine and an amino methyl group was also detected at both t_R_. These findings and fragmentation behavior correspond to previously published works [21, 22]. Based on the fragment *m/z* 238, we suggest that the hydroxy group is located on the aliphatic circle of SRT [21]. Both the SRT-OH isomers were found in the ISE and IRE strains in all homogenates. Mass *m/z* 322.08 at the both t_R_ with the same fragments was also noticeable in blank chemical samples, however, its intensity was approximately five times lower than in the samples. The intensity of *m/z* 322.08 detected in the medium was comparable with the chemical blank.

Other metabolites as two isomers of dihydroxy SRT (SRT-2OH), two isomers of SRT *O*-glucoside (SRT-*O*-GLC) and SRT ketone (SRT=O) were identified, but in a much lower intensity than SRT-OH. SRT-*O*-GLC *m/z* 484.13 [M + H]^+^, which results in the product ion *m/z* 273.02, suggests that glucose is connected to the hydroxy group on the aliphatic circle. Fragments *m/z* 176.09 and *m/z* 194.10 are supposed to be created by the shift of glucose to the nitrogen during fragmentation. Similar types of rearrangements were described by Fredenhagen, Kühnöl [23].

Fragmentation *m/z* 338.07 [M + H]^+^, which corresponds to SRT-2OH, produces product ions *m/z* 320.06 (NL of water) and 289.02 (loss of CH_3_NH_2_ from *m/z* 320.06). Product ions *m/z* 261.02 and 247.00 are created by cleavage of the aliphatic ring. Based on the fragments *m/*z 289, 261 and 247 we supposed the location of the hydroxyl groups to be on the aliphatic circle of SRT or on the nitrogen.

**Figure 5:**
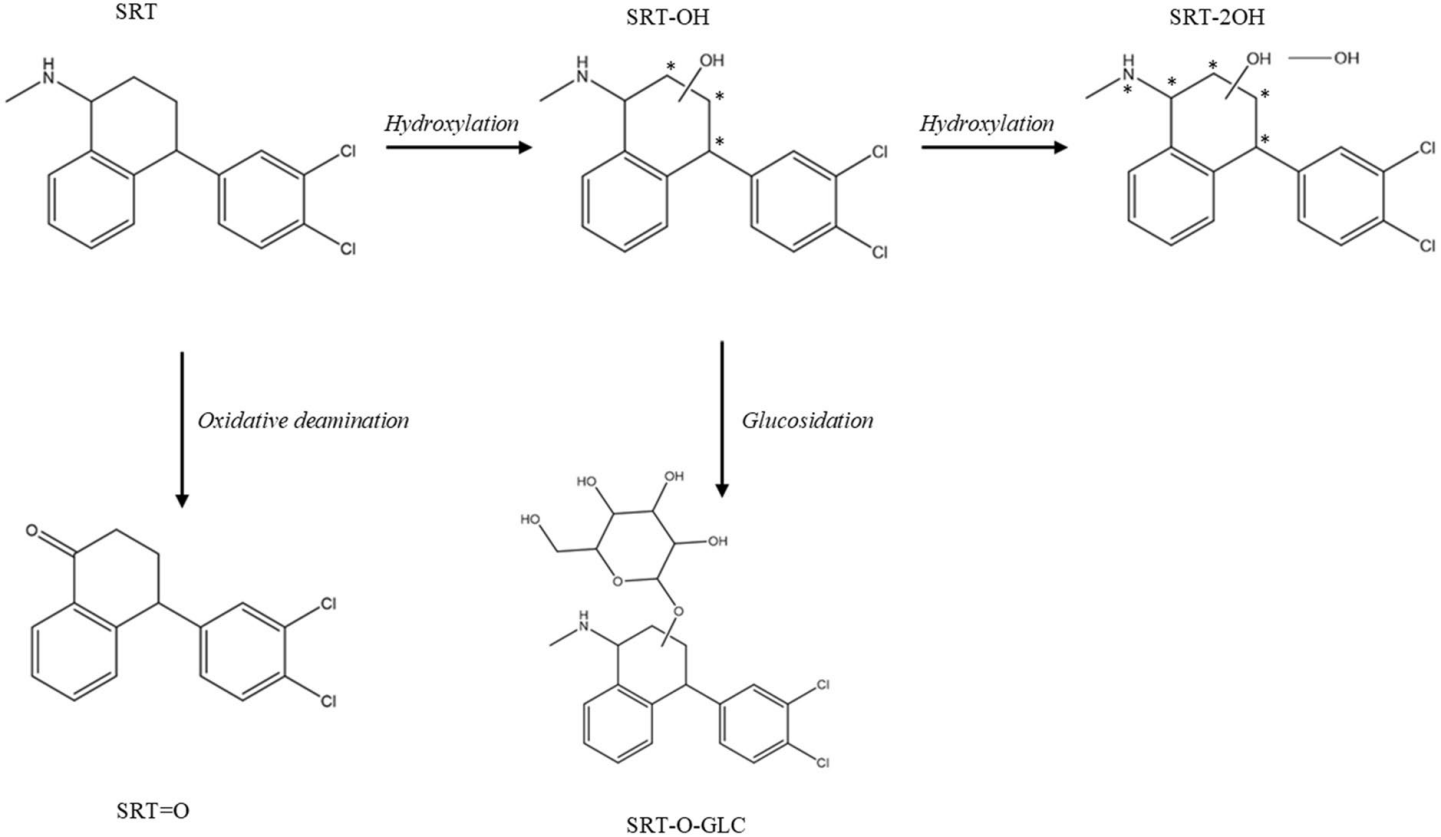
**The proposed metabolic pathway of SRT in *H. contortus* (ISE and IRE strain) adults.** The * marks possible location of the functional group.

Characterization of SRT = O was based on the transition of *m/z* 291.03 [M + H]^+^ to the two main fragments 273.02 and 238.05 and to the minor fragment 145.06. These fragments have been also previously reported [21, 22]. The proposed scheme of the SRT metabolic pathway in *H. contortus* is shown in Figure 5.

**Figure 6:**
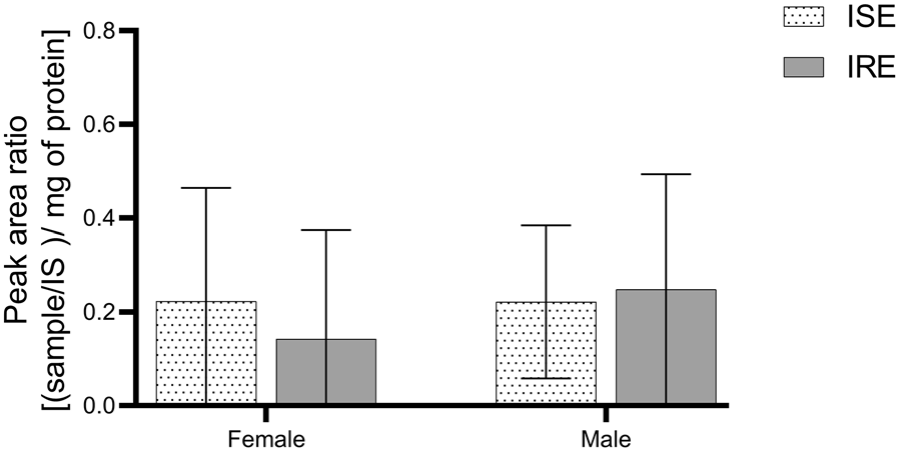
**Comparison of amount of hydroxyl metabolite (t**_**R**_** 10.48) of SRT in ISE and IRE strains of *H. contortus* adults.** Peak area ratio between sample and internal standard (IS) was normalized to mg of total protein. SA: Two-way ANOVA with Tukey’s multiple comparison test to compare strains and Šídák’s multiple comparison test to compare gender were used. Data are presented as means ± SD (*n* = 3).

To compare SRT biotransformation between the strains, the amount of the main SRT-OH in t_R_ 10.48 in each strain was semi-quantified. The low intensity of the other metabolites did not allow for their semi-quantification. The results (see Figure 6) showed no significant difference between the strains in the production of SRT-OH.

### Biotransformation of SRT in ovine liver

Precision-cut liver slices and a primary culture of hepatocytes were prepared from ovine liver and incubated with SRT (10 µM) for 24 h. A list of the metabolites including accurate masses, retention times and fragments of the metabolites is presented in Table 3. The extracted ion chromatograms, MS/MS fragmentation spectra and proposed structure of the product ions can be found in Additional files 11, 12, 13, 14, 15, 16, and 17.

**Table 3:** **List of the main metabolites, SRT and D3-SRT detected in the ovine liver samples with their retention times (t**_**R**_**) from LC–MS and LC-HRMS, m/z of precursor and product ions detected by LC-HRMS, elemental composition and designation**

Compound	Elemental composition	t_R_ LC–MS [min]	t_R_ LC-HRMS [min]	*m/z* precursor ions [M + H]^+^	*m/z* product ions [M + H]^+^	Designation
Desmethyl-SRT	C_16_H_15_Cl_2_N	4.73	12.02	292.0648	275.0388 158.9763 129.0702 91.0550	Desm-SRT
SRT	C_17_H_17_Cl_2_N	4.88	12.13	306.0815	275.0382 158.9758 129.0699 91.0548	SRT
SRT-D3 (IS)	C_17_H_17_Cl_2_N	4.88	12.13	309.0991	275.0394 158.9766 129.0699 91.0548	D3-SRT
Desmethyl-SRT-O-glucuronide	C_22_H_21_Cl_2_NO_7_	6.02 6.27	12.62 ^1^ 12.79 ^2^	482.0767	306.0449 ^1, 2^ 288.0343 ^1, 2^ 253.0654 ^1, 2^ 158.9763 ^1, 2^ 141.0182 ^1, 2^	Desm-SRT-O-GLU

Compared to *H. contortus*, in the ovine liver most of the SRT was metabolized. Two isomers of desmethyl *O*-glucuronides (desmSRT-*O*-GLU) with *m/z* 482.08 [M + H]^+^ at 12.62 and 12.79 min represented the main metabolites of SRT formed in the ovine liver. The product ion *m/z* 306.04 corresponds to a typical neutral loss for glucuronides Δ 176 [24]. The product ion *m/z* 288.03 is a result of subsequent NL Δ 18 (H_2_O). Both product ions were preset at both retention times, however the fragment *m/z* 288 was the most dominant product ion at 12.62 min, and fragment 306 was the most dominant product ion at 12.79 min. The product ion 253.06 was presented only in t_R_ 12.62 min and is formed by loss of chlorine. The product ion *m/z* 158.98 corresponds to a fragment of SRT, and *m/z* 141.01 is a residue of glucuronide acid. Based on the fragments, we suggest that O-glucuronides bind to the aliphatic circle of SRT or to the nitrogen.

The product ions of *m/z* 292 [M + H] ( *m/z* 275.04, 158.99, 129.07, 91.05) are identical with the product ions of SRT; these results correspond to described fragmentation for desmethyl SRT (desmSRT) in previous work [21].

**Figure 7:**
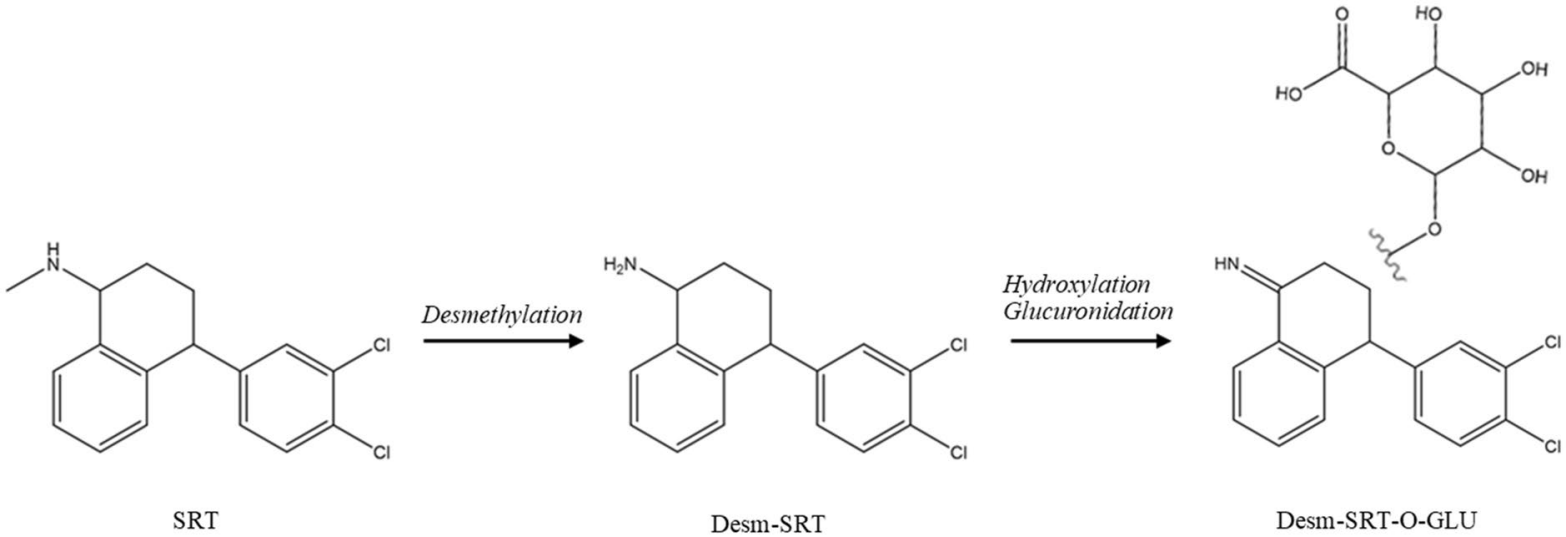
**The proposed metabolic pathway of SRT in ovine liver (liver slices and isolated hepatocytes).**

The scheme of the SRT metabolic pathway in ovine liver is presented in Figure 7.

## Discussion

The screening of drugs already approved for the treatment of other diseases and their review and possible repurposing for anthelmintic treatment represents an alternative to developing completely novel anthelmintic drugs. In addition to lower developmental costs, the advantage of drug repurposing, sometimes referred to as “therapeutic switching”, is the prior availability of preclinical and clinical data that might accelerate the drug approval process. Nevertheless, the major drawback of human drug repurposing for antiparasitic use in veterinary medicine is that this indication usually requires higher doses exceeding the ones tested during the toxicity studies for the previous registration, making it necessary to repeat tests with higher doses and in other species [25]. On the other hand, exploiting drugs from other indications may well serve as a promising approach in the effort to overcome drug resistance in target species of helminths. In addition, new anthelmintics might be found among drugs with a totally different structure which also increases the probability of different mechanism of action and efficacy in resistant strains of helminths [9].

Keiser et al. tested 1600 compounds from the FDA library, finding 12 substances to be effective against the L3 larvae of *Ancylostoma ceylanicum* [26]. Four of the 2745 compounds (either FDA-approved, launched or in clinical development) showed anthelmintic activity against exsheated L3 of *Cooperia oncophora* and appeared as promising candidates for further studies [27]. A study by Weeks et al. uncovered the anthelmintic activity of the neuromodulatory drugs SRT, paroxetine and chlorpromazine, with SRT proving the most effective. The identification of a different mechanism of action in these drugs than that of other anthelmintics on the market seemed to be quite promising. Moreover, this mechanism is different from the anti-depressant or anti-psychotic effects in humans associated with SRT, which decreases the risk of undesirable neurological side effects in hosts [10]. As the anthelmintic effect of SRT had not been previously tested in *H. contortus*, we decided to fill this research gap with our study.

Firstly, *H. contortus* eggs were used to evaluate the ability of SRT to inhibit egg hatching. Although SRT had previously been found to impair the hatching of *Ancylostoma caninum* eggs [10], no effect of SRT was observed in *H. contortus* eggs*.* However, most of anthelmintics (with exception of benzimidazoles) are not ovicidal and their toxicity to parasitic stages of helminths is the principle of their efficacy in treatment. Thus in our work, *H. contortus* adults were exposed to SRT and the effect was evaluated using a newly developed ATP bioluminescent assay [13]. To our knowledge, this is the only available biochemical method targeting the parasitic stage which causes haemonchosis, i.e. adult worms. The other advantage of this method is its sensitivity, hence the low amounts of biological material are necessary [13]. Using this method, the effect of SRT was tested in females and males of *H. contortus* separately. In the drug-sensitive ISE strain, SRT decreased the viability of both genders, with males proving more sensitive. SRT IC_50_ 14.8 and 3.7 µM were calculated in the females and males, respectively. The higher sensitivity of the males than females of *H. contortus* to the common anthelmintics LEV and MOP was observed in the previous study [13], but the reason for this remains unclear. In the results of a study by Weeks et al. for the free-living nematode *Caenorhabditis elegans* the IC_50_ of SRT was 18.2 µM, while in the parasitic nematodes *Trichuris muris* and *Schistosoma mansoni* the SRT IC_50_ were 7.2 and 8.4 µM, respectively [10]. As a mixture of both genders was used in these experiments [10], the data are in a good agreement with those obtained in *H. contortus* if the values obtained in females and males are averaged. In terms of comparisons to other anthelmintics, we found no significant differences between the effect of SRT and classical anthelmintics LEV and MOP in *H. contortus* adults of the ISE strain.

Although SRT efficacy in drug-sensitive ISE is important, its efficacy in the drug-resistant strain of *H. contortus* would prove more beneficial. Therefore, the effect of SRT was also studied in drug-resistant IRE. In males of the IRE strain, SRT showed a similar effect to that of the males of the ISE strain. In females, however, lower efficacy of SRT in the IRE strain than in the ISE strain was observed. The rising ATP level in females of the IRE strain could be explained as reaction to stress caused by the presence of SRT [28]. In any case, certain efficacy of SRT was also proved in the resistant IRE strain. These results indicate SRT as potential candidate for haemonchosis treatment.

Nevertheless, the use of SRT for haemonchosis treatment requires its nontoxicity in sheep as the main target species. Therefore, the effect of SRT on ovine liver was tested using two in vitro models: precision-cut liver slices and a primary culture of hepatocytes. As SRT did not show hepatotoxicity up to 75 µM concentration in these models, it can be assumed that SRT at an anthelmintically effective concentration is not toxic to ovine liver. It will obviously be necessary to exclude the in vivo toxicity of SRT in sheep.

In the next part of our project, the biotransformation of SRT was tested to reveal the ability of this parasite to protect against SRT via its deactivation. As sheep is the intended target species, SRT biotransformation was also studied in ovine liver. *H. contortus* was not shown to metabolize sertraline very extensively and most of the parent drug remained unmetabolized. Two positional isomers of hydroxy SRT (SRT-OH) were the main metabolites, while only traces of other metabolites such as SRT-*O*-glucoside, dihydroxy-SRT, and SRT-ketone were found in *H. contorts* adults. When metabolism of SRT was studied in the protozoan *Spirostomum ambiguum* [29], weak biotransformation was also observed. Hydroxy-SRT and other metabolites with non-identified structures were detected [29]. The weak biotransformation in *H. contortus* is a definite positive finding in our SRT experiments, as this indicates that *H. contortus* is not able to effectively defend against SRT via its deactivation. Moreover, when the amount of SRT-OH formed in *H. contortus* was semi-quantified and compared among strains no differences were observed.

Previously, the biotransformation of benzimidazole anthelmintics has been studied in *H. contortus* adults. Drug-resistant strains of the nematodes metabolized these anthelmintics much more effectively than ISE strain and the increased biotransformation is considered as one of resistance mechanisms [30]. In case of SRT however, its milder effect in IRE than ISE females was not based on increased SRT biotransformation in the IRE strains. Compared to *H. contortus*, ovine liver metabolized SRT much more extensively and in different way, mainly via desmethylation and glucuronidation. *N*-desmethyl-SRT is the major human metabolite of SRT. In addition, other metabolites such as hydroxy-SRT, N-hydroxy-SRT, SRT-ketone and their glucuronides are formed in humans [31, 32].

In conclusion, SRT in micromolar concentrations decrease viability of *H. contortus* adults from both the drug-sensitive ISE strain and the drug-resistant IRE strain. At these concentrations SRT is not toxic to ovine liver. *H. contortus* is not able to protect itself against SRT through its extensive biotransformation. Therefore, SRT as a potential drug against haemonchosis should certainly be tested further.

## Supplementary Information

**Additional file 1. Extracted ion UHPLC-HRMS chromatograms of SRT-OH (m/z 322.0760) from A) sample (*H. contortus* female ISE incubated with SRT) B) biological blank and C) chemical blank.** D) HRMS/MS spectrum of SRT-OH in t_R_ 10.49. E) HRMS/MS spectrum of SRT-OH in t_R_ 11.36.

**Additional file 2. Comparison of m/z of SRT-OH and its fragments calculated by Mass Frontier software with our measured masses and proposed fragment structure.**

**Additional file 3. Extracted ion UHPLC-HRMS chromatograms of SRT-O-GLC (m/z 484.1285) from A) sample (*H. contortus* female ISE incubated with SRT) B) biological blank and C) chemical blank.** D) HRMS/MS spectrum SRT-O-GLC in t_R_ 9.53. E) HRMS/MS spectrum SRT-O-GLC in t_R_ 10.66.

**Additional file 4. Comparison of m/z of SRT-O-GLC and its fragments calculated by Mass Frontier software with our measured masses and proposed fragment structure.**

**Additional file 5. Extracted ion UHPLC-HRMS chromatograms of SRT-2OH (m/z 338.0717) from A) sample (*H. contortus* female ISE incubated with SRT) B) biological blank and C) chemical blank**. D) HRMS/MS spectrum SRT-2OH in t_R_ 9.75. E) HRMS/MS spectrum SRT-2OH in t_R_ 10.04.

**Additional file 6. Comparison of m/z of SRT-2OH and its fragments calculated by Mass Frontier software with our measured masses and proposed fragment structure.**

**Additional file 7. Extracted ion UHPLC-HRMS chromatograms of SRT = O (m/z 291.0338) from A) sample (*H. contortus* female ISE incubated with SRT) B) biological blank and C) chemical blank.** D) HRMS/MS spectrum of SRT = O.

**Additional file 8. Comparison of m/z SRT = O and its fragments calculated by Mass Frontier software with our measured masses and proposed fragment structure.**

**Additional file 9. Comparison of UHPLC-MS chromatograms of sample (*H. contortus* female IRE incubated with SRT) with chemical blank (Blank CH) and biological blank (Blank B).** Identified metabolite SRT-OH was detected in the sample and in small intensity in the chemical blank and was not found in the biological blank.

**Additional file 10. Comparison of UHPLC-MS chromatograms of sample (*H. contortus* female IRE incubated with SRT) with chemical blank (Blank CH) and biological blank (Blank B).** Identified metabolites SRT-2OH, SRT-O-GLC and SRT = O were detected in the sample and were not found in blank samples.

**Additional file 11. Extracted ion UHPLC-HRMS chromatograms of SRT (m/z 306.0815) from A) sample (isolated hepatocytes incubated with SRT) B) biological blank and C) chemical blank.** D) HRMS/MS spectrum of SRT.

**Additional file 12. Comparison of m/z of SRT and its fragments calculated by Mass Frontier software with our measured masses and proposed fragment structure.**

**Additional file 13. Extracted ion UHPLC-HRMS chromatograms of Desm-SRT (m/z 292.0648) from A) sample (isolated hepatocytes incubated with SRT) B) biological blank and C) chemical blank.** D) HRMS/MS spectrum of Desm-SRT.

**Additional file 14. Comparison of m/z of Desm-SRT and its fragments calculated by Mass Frontier software with our measured masses and proposed fragment structure.**

**Additional file 15. Extracted ion UHPLC-HRMS chromatograms of Desm-SRT-O-GLU (m/z 482.0767) from A) sample (isolated hepatocytes incubated with SRT) B) biological blank and C) chemical blank.** D) HRMS/MS spectrum of Desm-SRT-O-GLU in t_R_ 12.63. E) HRMS/MS spectrum of Desm-SRT-O-GLU in t_R_ 12.79 min.

**Additional file 16. Comparison of m/z of Desm-SRT-O-GLU and its fragments calculated by Mass Frontier software with our measured masses and proposed fragment structure.**

**Additional file 17. Comparison of UHPLC-MS chromatograms of sample (hepatocytes cells or medium) with biological blank (Blank B) and chemical blank (Blank CH).** Identified metabolites Desm-SRT) and Desm-SRT-O-GLU were found in samples of media and cells but was not detected in blank samples.

## Data Availability

The datasets supporting the conclusions of this article are included within the article text and additional files.

## Abbreviations

ACN: acetonitrile
ATP: adenosine triphosphate
BCA: bicinchoninic acid
BSA: bovine serum albumin
DMSO: dimethyl sulfoxide
EDTA: ethylenediaminetetraacetic acid
EGTA: ethylene glycol-bis(β-aminoethyl ether)-*N*,*N*,*N*′,*N*′-tetra acetic acid
FBS: fetal bovine serum
ISE: inbred susceptible-Edinburgh strain (MHco3)
IVM: ivermectin
LEV: levamisole
MOP: monepantel
MTT: (3-(4,5-dimethylthiazol-2-yl)-2,5-diphenyltetrazolium bromide)
PBS: phosphate-buffered saline
SRT: sertraline
Tris: tris(hydroxymethyl)aminomethane
