# Supplementary figures and images for: Sertraline as a new potential anthelmintic against Haemonchus contortus: toxicity, efficacy, and biotransformation
Source: Vet Res. 2021 Dec 11;52:143. doi: 10.1186/s13567-021-01012-x (PMC8666012; doi:10.1186/s13567-021-01012-x)

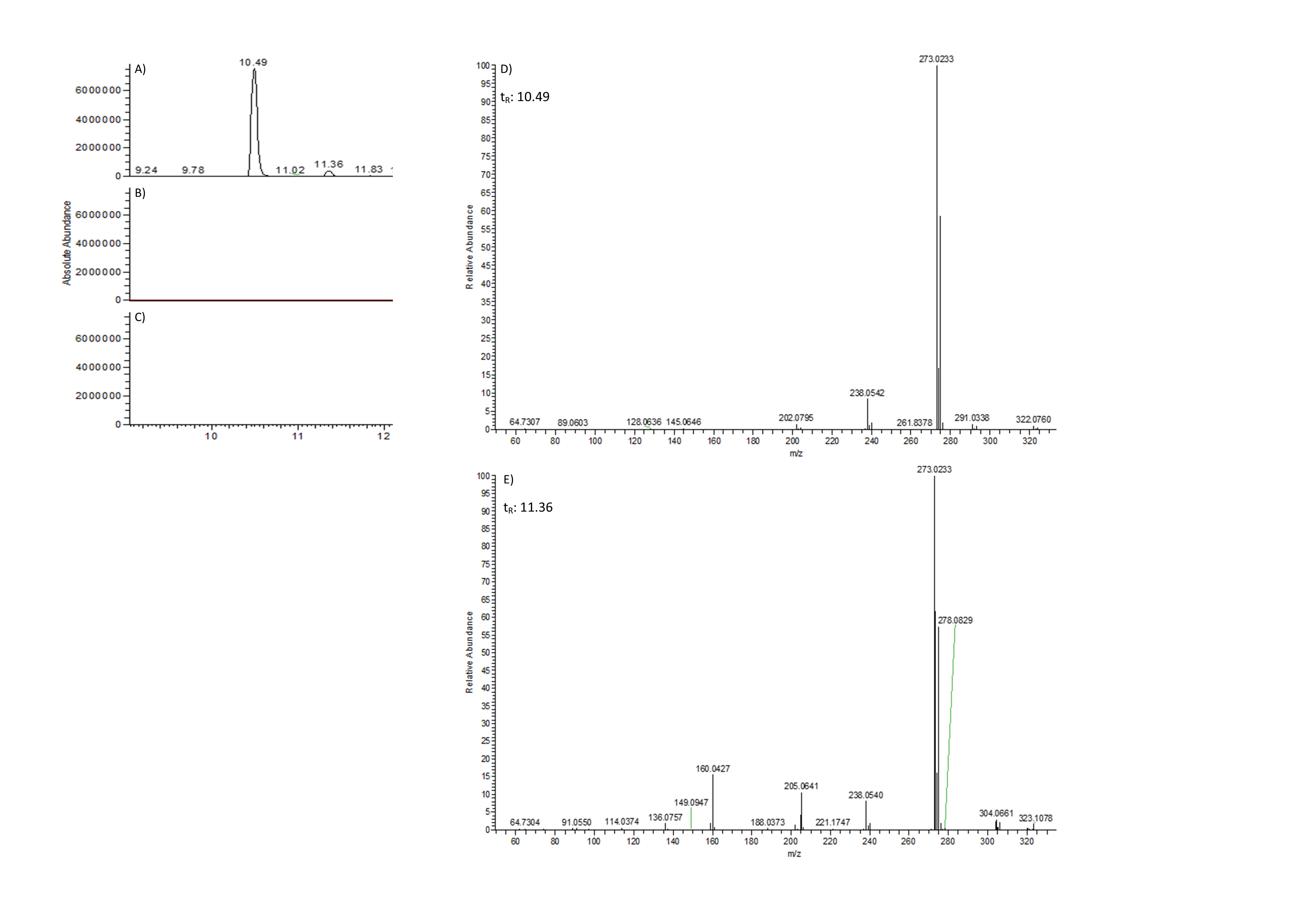

Supplement: Supplementary file 1 — Additional file 1. Extracted ion UHPLC-HRMS chromatograms of SRT-OH (m/z 322.0760) from A) sample (H. contortus female ISE incubated with SRT) B) biological blank and C) chemical blank. D) HRMS/MS spectrum of SRT-OH in tR 10.49. E) HRMS/MS spectrum of SRT-OH in tR 11.36. [file 13567_2021_1012_MOESM1_ESM.tif]

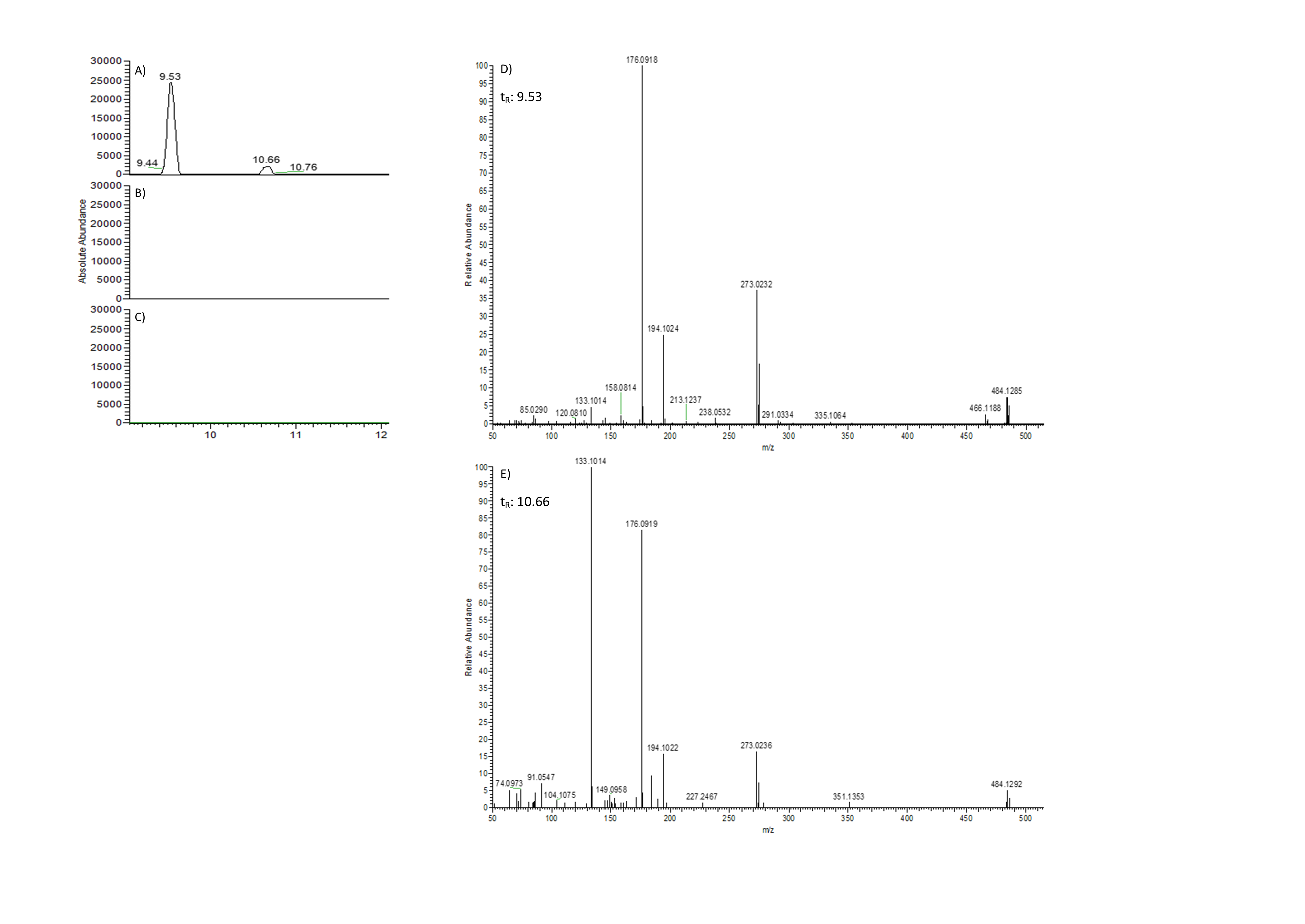

Supplement: Supplementary file 3 — Additional file 3. Extracted ion UHPLC-HRMS chromatograms of SRT-O-GLC (m/z 484.1285) from A) sample (H. contortus female ISE incubated with SRT) B) biological blank and C) chemical blank. D) HRMS/MS spectrum SRT-O-GLC in tR 9.53. E) HRMS/MS spectrum SRT-O-GLC in tR 10.66. [file 13567_2021_1012_MOESM3_ESM.tif]

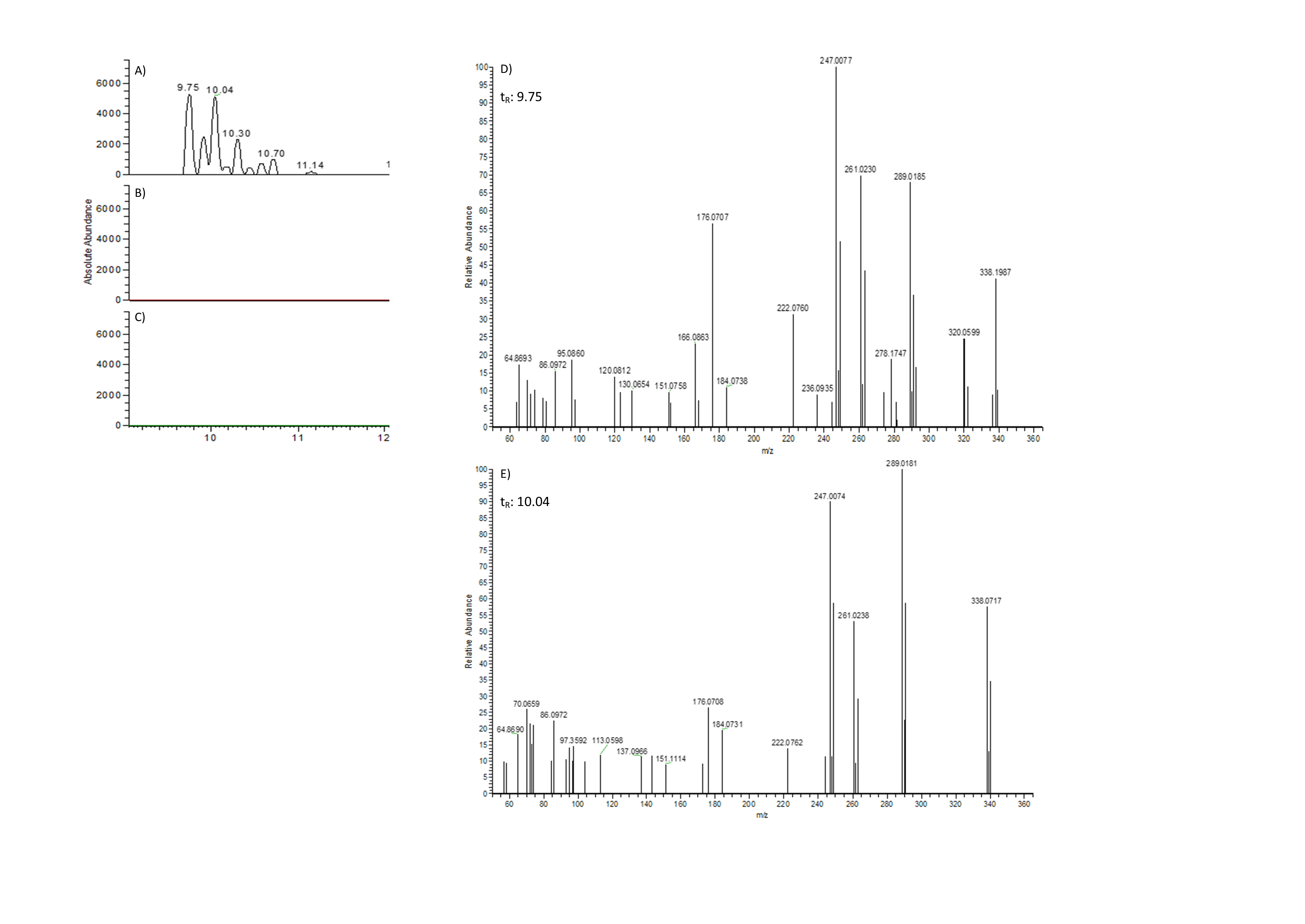

Supplement: Supplementary file 5 — Additional file 5. Extracted ion UHPLC-HRMS chromatograms of SRT-2OH (m/z 338.0717) from A) sample (H. contortus female ISE incubated with SRT) B) biological blank and C) chemical blank. D) HRMS/MS spectrum SRT-2OH in tR 9.75. E) HRMS/MS spectrum SRT-2OH in tR 10.04. [file 13567_2021_1012_MOESM5_ESM.tif]

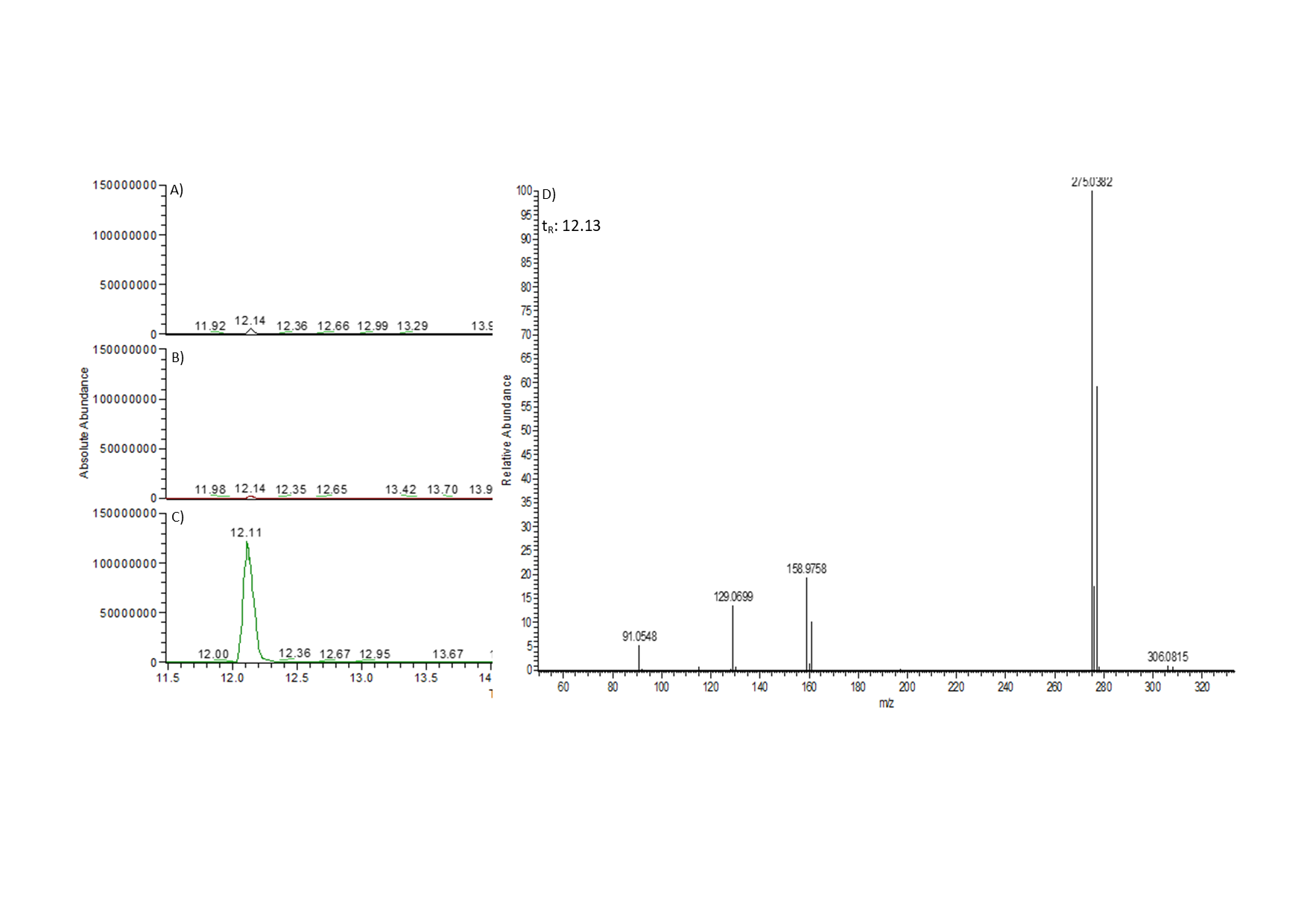

Supplement: Supplementary file 7 — Additional file 7. Extracted ion UHPLC-HRMS chromatograms of SRT = O (m/z 291.0338) from A) sample (H. contortus female ISE incubated with SRT) B) biological blank and C) chemical blank. D) HRMS/MS spectrum of SRT = O. [file 13567_2021_1012_MOESM7_ESM.tif]

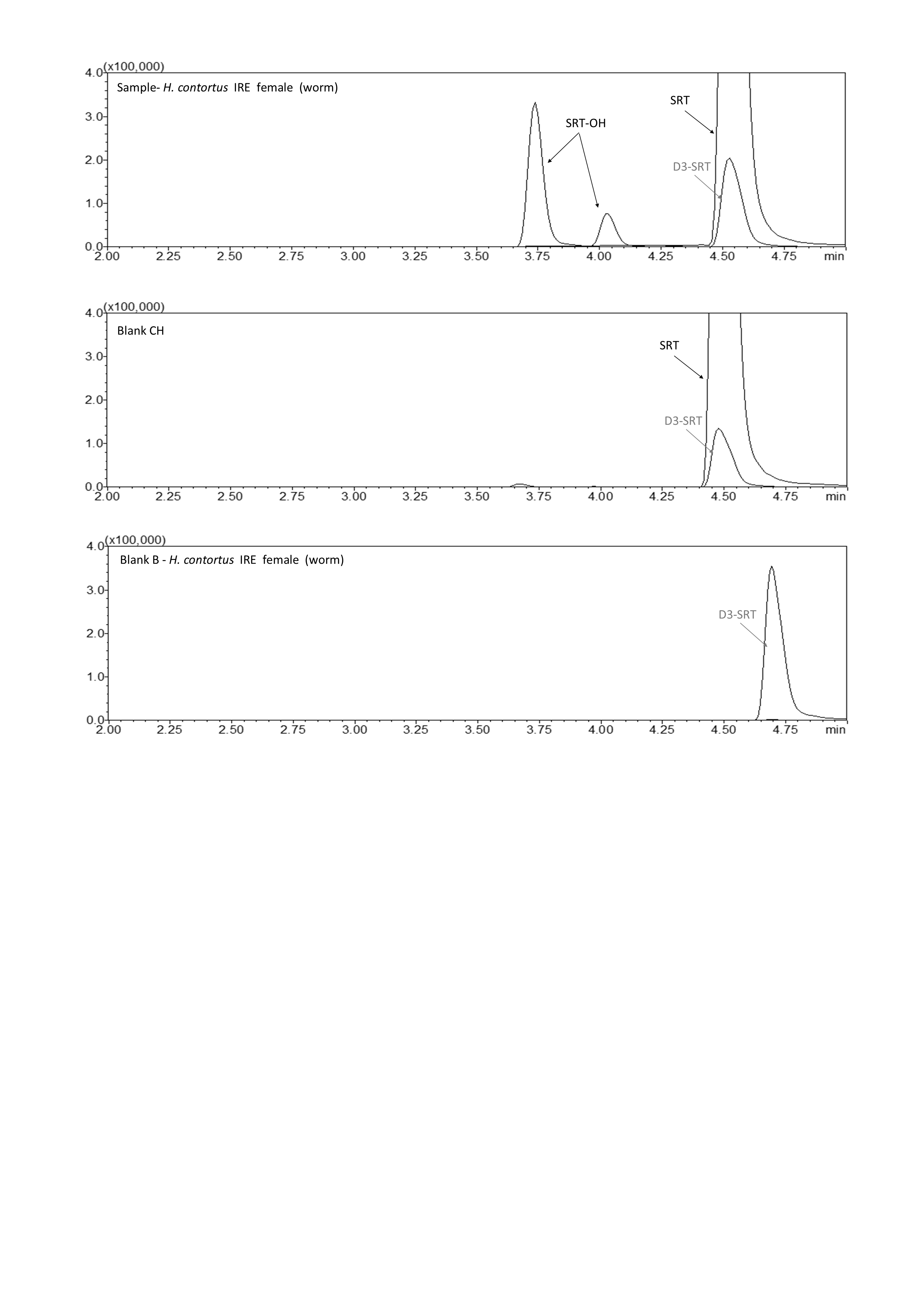

Supplement: Supplementary file 9 — Additional file 9. Comparison of UHPLC-MS chromatograms of sample (H. contortus female IRE incubated with SRT) with chemical blank (Blank CH) and biological blank (Blank B). Identified metabolite SRT-OH was detected in the sample and in small intensity in the chemical blank and was not found in the biological blank. [file 13567_2021_1012_MOESM9_ESM.tif]

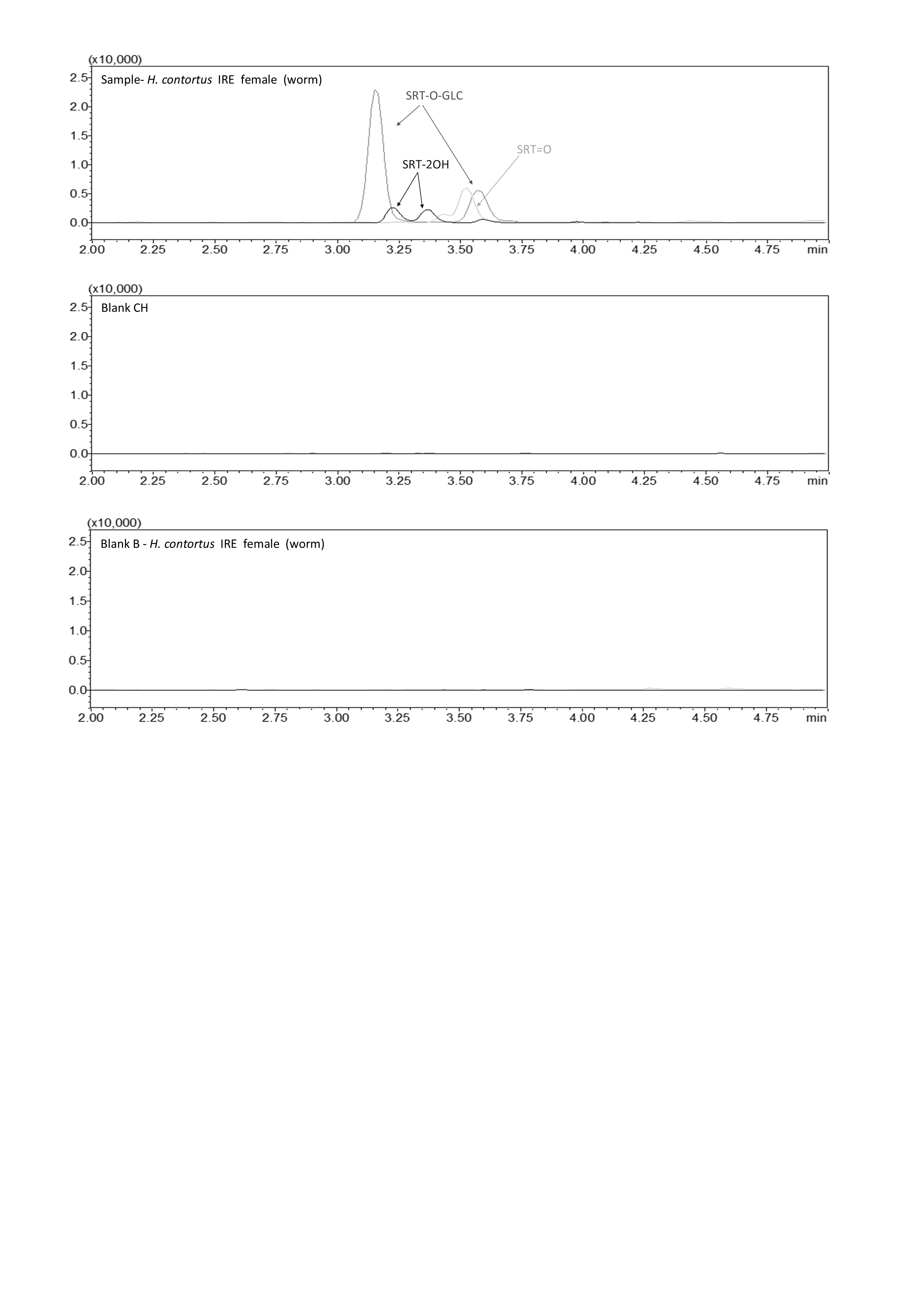

Supplement: Supplementary file 10 — Additional file 10. Comparison of UHPLC-MS chromatograms of sample (H. contortus female IRE incubated with SRT) with chemical blank (Blank CH) and biological blank (Blank B). Identified metabolites SRT-2OH, SRT-O-GLC and SRT = O were detected in the sample and were not found in blank samples. [file 13567_2021_1012_MOESM10_ESM.tif]

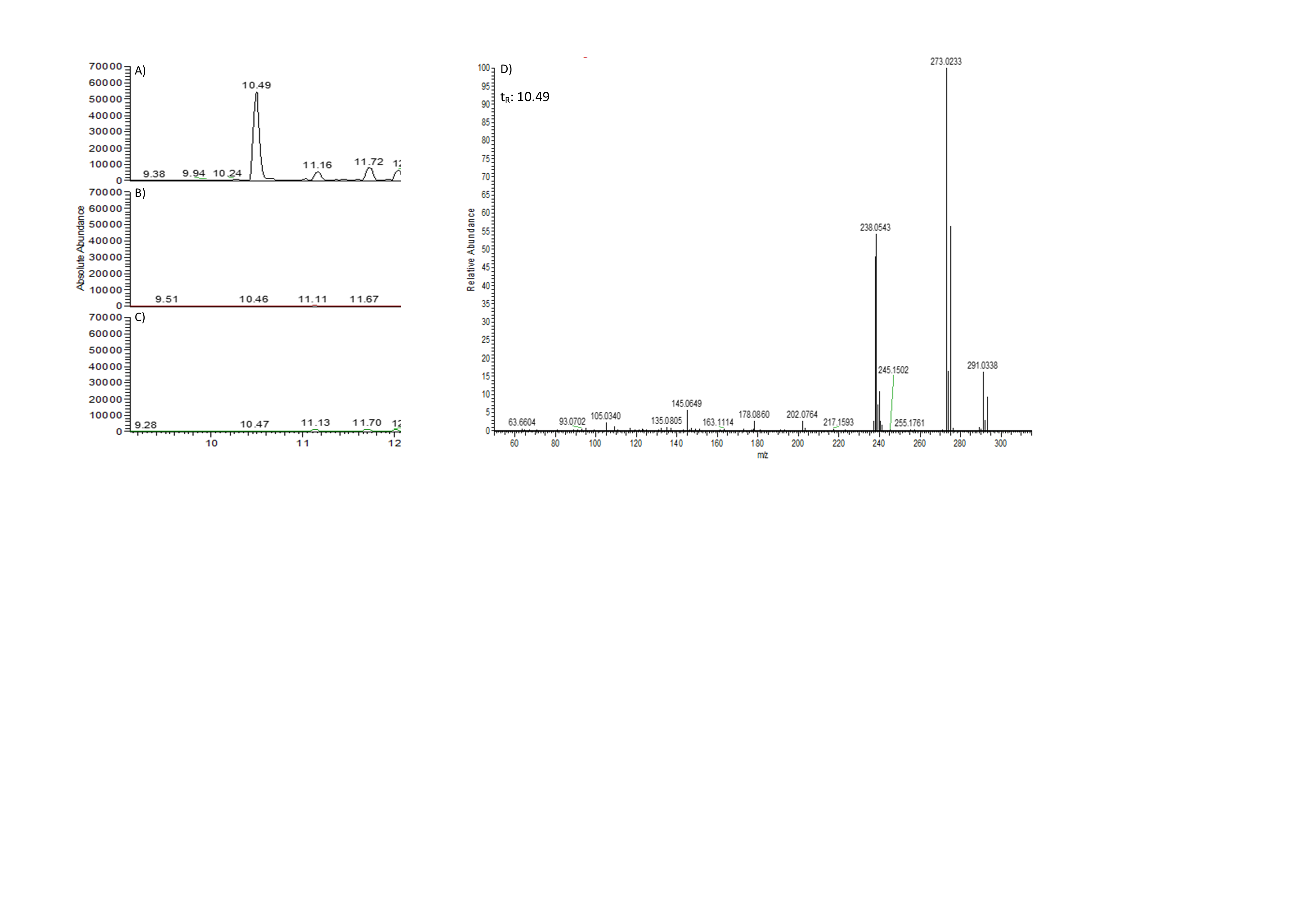

Supplement: Supplementary file 11 — Additional file 11. Extracted ion UHPLC-HRMS chromatograms of SRT (m/z 306.0815) from A) sample (isolated hepatocytes incubated with SRT) B) biological blank and C) chemical blank. D) HRMS/MS spectrum of SRT. [file 13567_2021_1012_MOESM11_ESM.tif]

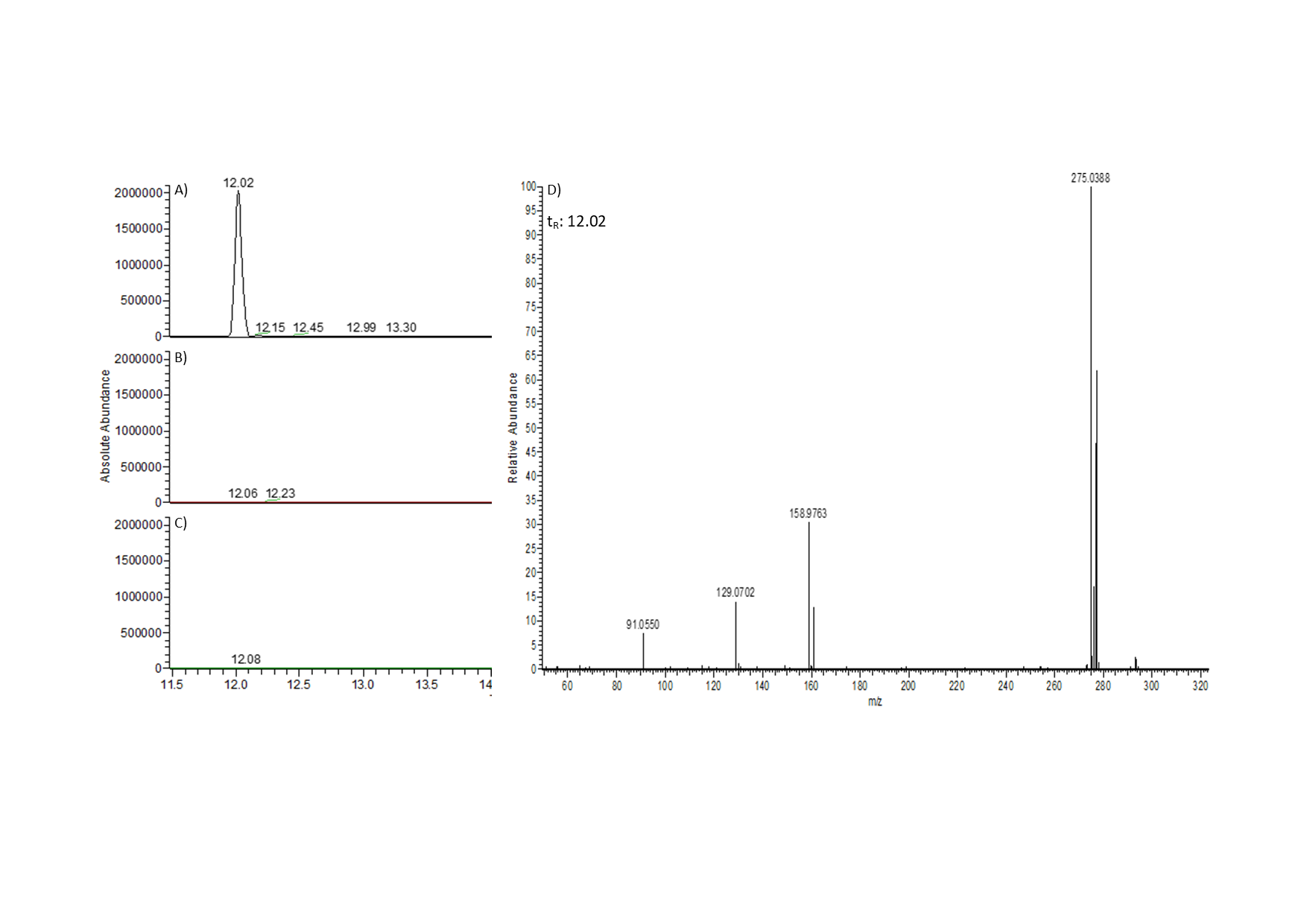

Supplement: Supplementary file 13 — Additional file 13. Extracted ion UHPLC-HRMS chromatograms of Desm-SRT (m/z 292.0648) from A) sample (isolated hepatocytes incubated with SRT) B) biological blank and C) chemical blank. D) HRMS/MS spectrum of Desm-SRT. [file 13567_2021_1012_MOESM13_ESM.tif]

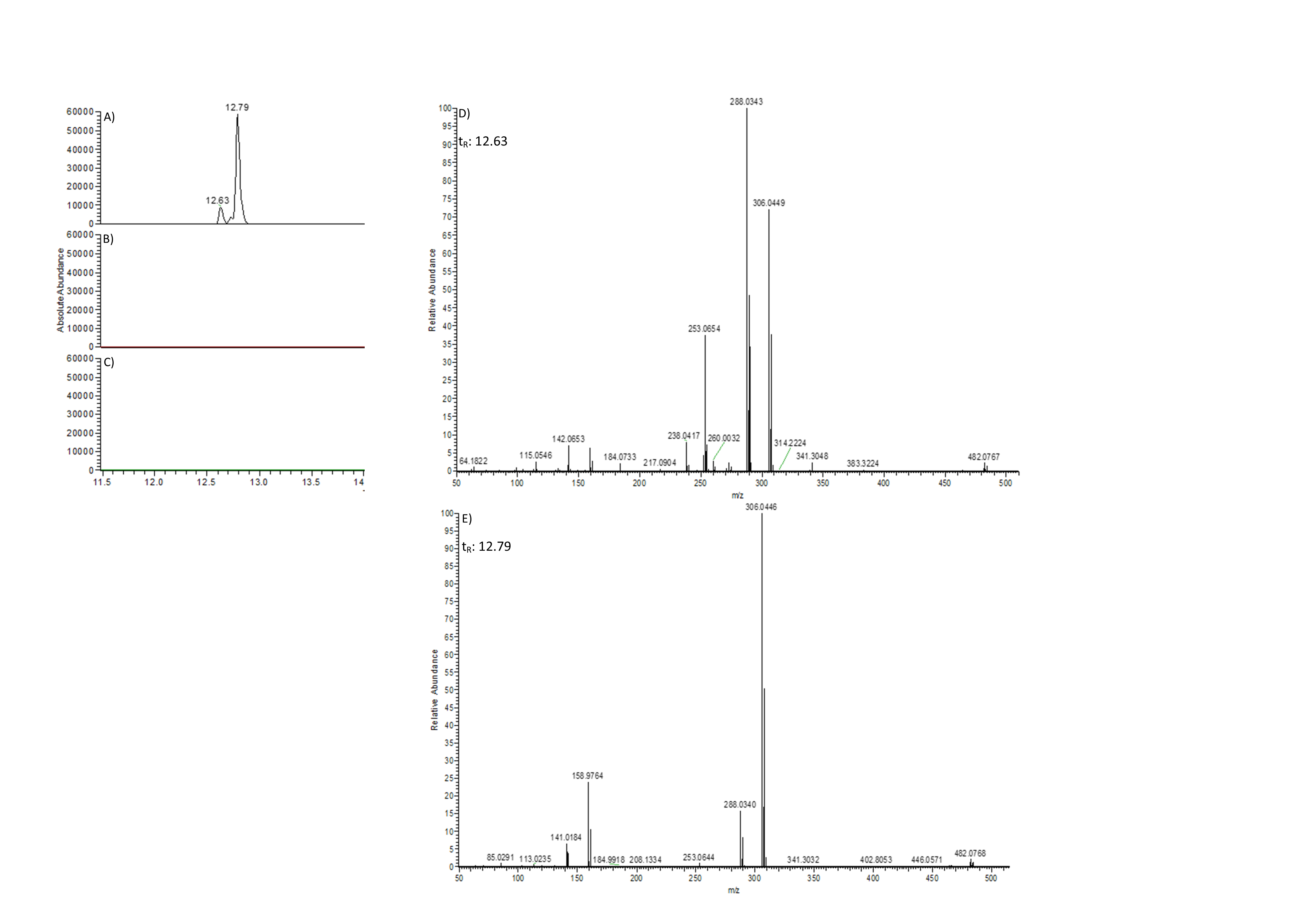

Supplement: Supplementary file 15 — Additional file 15. Extracted ion UHPLC-HRMS chromatograms of Desm-SRT-O-GLU (m/z 482.0767) from A) sample (isolated hepatocytes incubated with SRT) B) biological blank and C) chemical blank. D) HRMS/MS spectrum of Desm-SRT-O-GLU in tR 12.63. E) HRMS/MS spectrum of Desm-SRT-O-GLU in tR 12.79 min. [file 13567_2021_1012_MOESM15_ESM.tif]

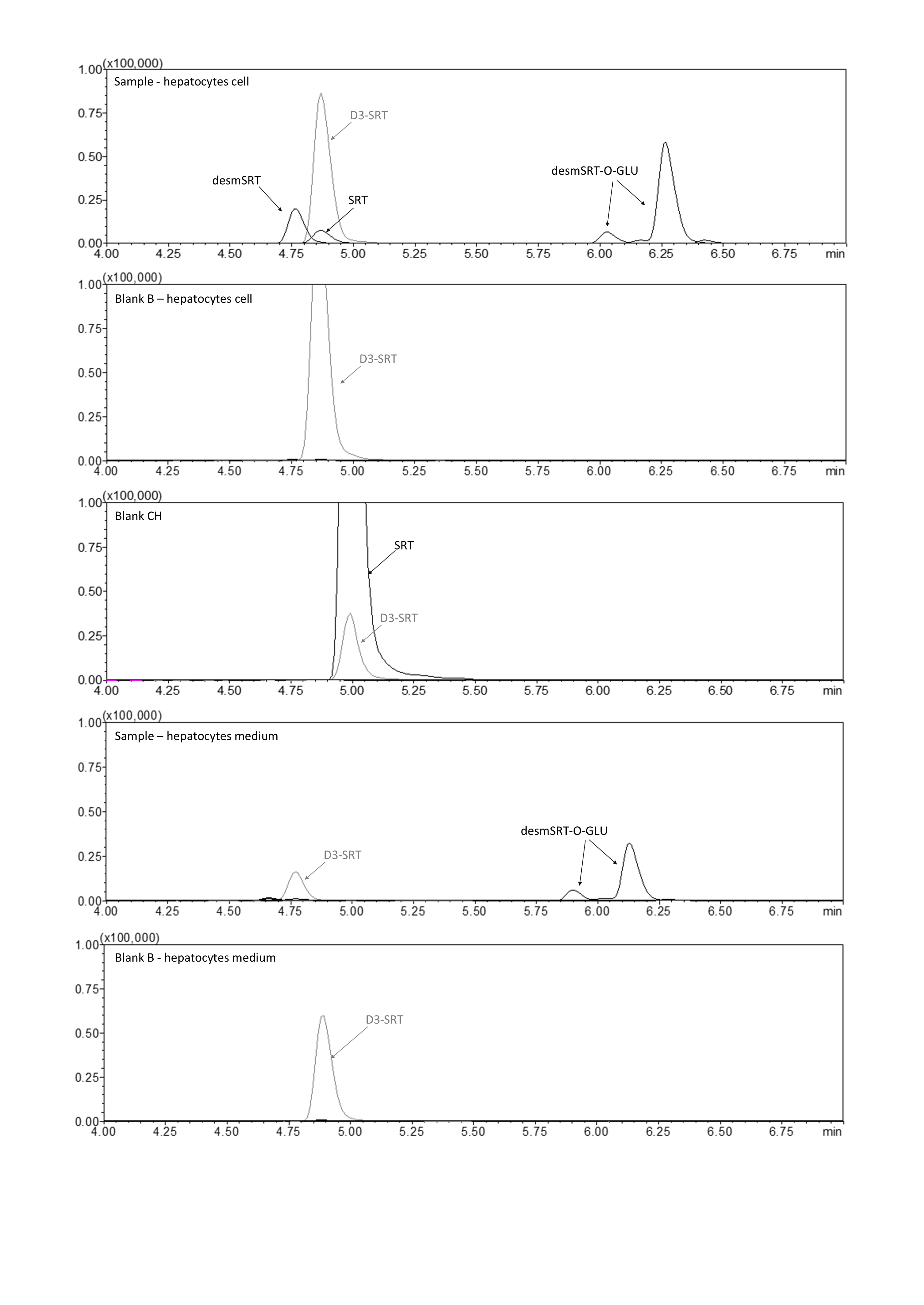

Supplement: Supplementary file 17 — Additional file 17. Comparison of UHPLC-MS chromatograms of sample (hepatocytes cells or medium) with biological blank (Blank B) and chemical blank (Blank CH). Identified metabolites Desm-SRT) and Desm-SRT-O-GLU were found in samples of media and cells but was not detected in blank samples. [file 13567_2021_1012_MOESM17_ESM.tif]
