# Supplementary material for: Sertraline as a new potential anthelmintic against Haemonchus contortus: toxicity, efficacy, and biotransformation
Source: Vet Res. 2021 Dec 11;52:143. doi: 10.1186/s13567-021-01012-x (PMC8666012; doi:10.1186/s13567-021-01012-x)
Supplement: Supplementary file 4 — Additional file 4. Comparison of m/z of SRT-O-GLC and its fragments calculated by Mass Frontier software with our measured masses and proposed fragment structure. [file 13567_2021_1012_MOESM4_ESM.docx]

**Additional file 4 Comparison of m/z of SRT-O-GLC and its fragments calculated by Mass Frontier software with our measured masses and proposed fragment structure**

| Fragment | 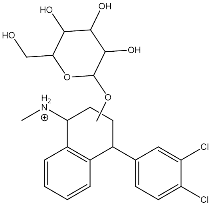 | 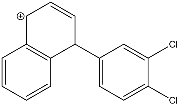 | 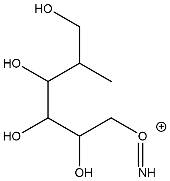 | 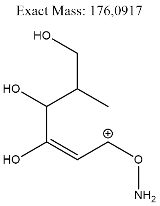 |
| --- | --- | --- | --- | --- |
| Calculated Mass  [M+H]^+^ | 484.1288 | 273.0232 | 194.1023 | 176.0917 |
| Measured Mass  [M+H]^+^ | 484.1285 | 273.0232 | 194.1024 | 176.0918 |
