# Supplementary material for: Sertraline as a new potential anthelmintic against Haemonchus contortus: toxicity, efficacy, and biotransformation
Source: Vet Res. 2021 Dec 11;52:143. doi: 10.1186/s13567-021-01012-x (PMC8666012; doi:10.1186/s13567-021-01012-x)
Supplement: Supplementary file 6 — Additional file 6. Comparison of m/z of SRT-2OH and its fragments calculated by Mass Frontier software with our measured masses and proposed fragment structure. [file 13567_2021_1012_MOESM6_ESM.docx]

**Additional file 6 Comparison of m/z of SRT-2OH and its fragments calculated by Mass Frontier software with our measured masses and proposed fragment structure**

| Fragment | 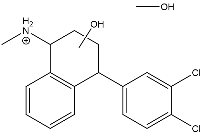 | 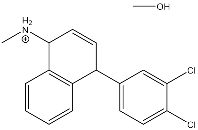 | 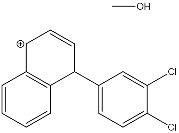 | 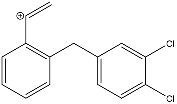 | 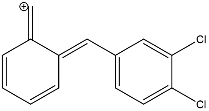 |
| --- | --- | --- | --- | --- | --- |
| Calculated Mass [M+H]^+^ | 338.0709 | 320.0603 | 289.0181 | 261.0232 | 247.0076 |
| Measured Mass [M+H]^+^ | 338.0717 | 320.0599 | 289.0185 | 261.0230 | 247.0077 |
