# Supplementary material for: Sertraline as a new potential anthelmintic against Haemonchus contortus: toxicity, efficacy, and biotransformation
Source: Vet Res. 2021 Dec 11;52:143. doi: 10.1186/s13567-021-01012-x (PMC8666012; doi:10.1186/s13567-021-01012-x)
Supplement: Supplementary file 8 — Additional file 8. Comparison of m/z SRT = O and its fragments calculated by Mass Frontier software with our measured masses and proposed fragment structure. [file 13567_2021_1012_MOESM8_ESM.docx]

**Additional file 8 Comparison of m/z SRT=O and its fragments calculated by Mass Frontier software with our measured masses and proposed fragment structure**

| Fragment | 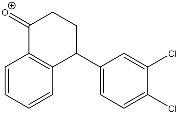 | 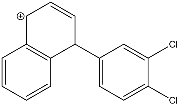 | 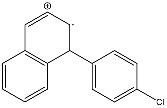 |
| --- | --- | --- | --- |
| Calculated Mass [M+H]^+^ | 291.0338 | 273.0232 | 238.0544 |
| Measured Mass [M+H]^+^ | 291.0338 | 273.0233 | 238.0543 |
