# Supplementary material for: Sertraline as a new potential anthelmintic against Haemonchus contortus: toxicity, efficacy, and biotransformation
Source: Vet Res. 2021 Dec 11;52:143. doi: 10.1186/s13567-021-01012-x (PMC8666012; doi:10.1186/s13567-021-01012-x)
Supplement: Supplementary file 12 — Additional file 12. Comparison of m/z of SRT and its fragments calculated by Mass Frontier software with our measured masses and proposed fragment structure. [file 13567_2021_1012_MOESM12_ESM.docx]

**Additional file 12 Comparison of m/z of SRT and its fragments calculated by Mass Frontier software with our measured masses and proposed fragment structure.**

|  | 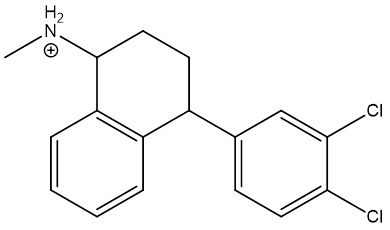 | 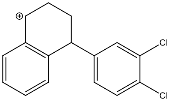 | 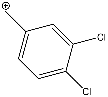 | 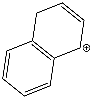 | 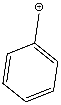 |
| --- | --- | --- | --- | --- | --- |
| Calculated Mass [M+H]^+^ | 306.0811 | 275.0389 | 158.9763 | 129.0699 | 91.0542 |
| Measured Mass [M+H]^+^ | 306.0815 | 275.0382 | 158.9758 | 129.0699 | 91.0548 |
