# Supplementary material for: Sertraline as a new potential anthelmintic against Haemonchus contortus: toxicity, efficacy, and biotransformation
Source: Vet Res. 2021 Dec 11;52:143. doi: 10.1186/s13567-021-01012-x (PMC8666012; doi:10.1186/s13567-021-01012-x)
Supplement: Supplementary file 16 — Additional file 16. Comparison of m/z of Desm-SRT-O-GLU and its fragments calculated by Mass Frontier software with our measured masses and proposed fragment structure. [file 13567_2021_1012_MOESM16_ESM.docx]

**Additional file 16 Comparison of m/z of Desm-SRT-O-GLU and its fragments calculated by Mass Frontier software with our measured masses and proposed fragment structure.**

| Fragment | 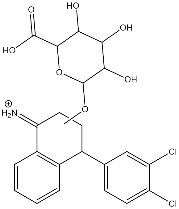 | 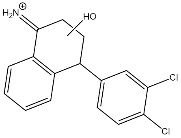 | 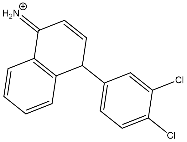 | 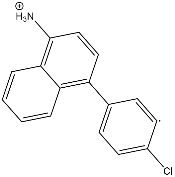 | 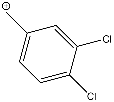 | 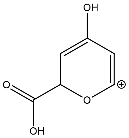 |
| --- | --- | --- | --- | --- | --- | --- |
| Calculated Mass  [M+H]^+^ | 482.0768 | 306.0447 | 288.0341 | 253.0653 | 158.9763 | 141.0182 |
| Measured Mass  [M+H]^+^ | 482.0767 | 306.0449 | 288.0343 | 253.0654 | 158.9764 | 141.0182 |
